# Influence of Cage Effects in Directing the Outcome of C–X Bond Forming Reactions

**DOI:** 10.1021/acsorginorgau.3c00044

**Published:** 2023-10-25

**Authors:** Zihang Qiu, Constanze N. Neumann

**Affiliations:** Department of Heterogeneous Catalysis, Max-Planck Institut für Kohlenforschung, Kaiser-Wilhelm-Platz 1, 45470 Mülheim an der Ruhr, Germany

**Keywords:** cage effect, radical pair, spin state, cage escape, solvent cage

## Abstract

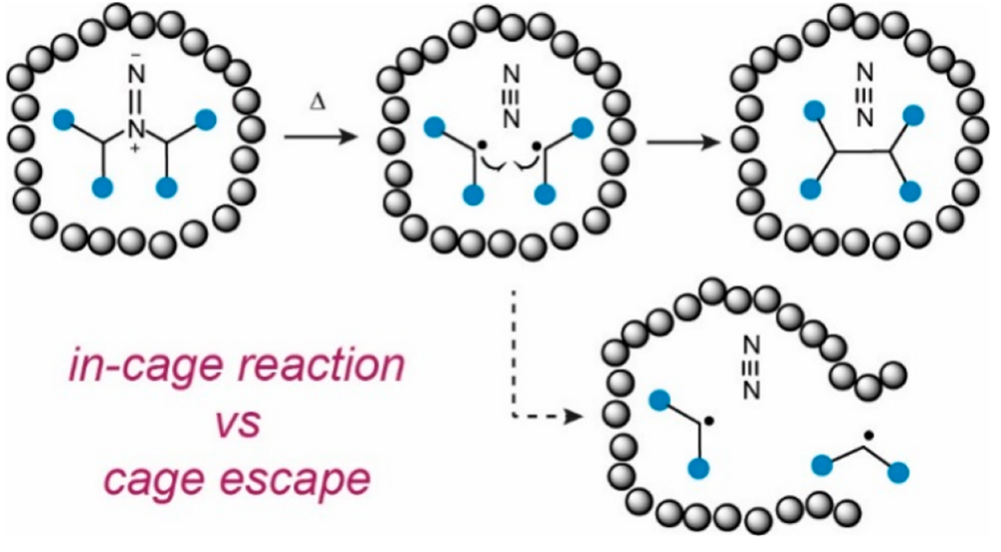

Radical reactions have recently experienced a resurgence in organic chemistry after many decades of being considered to be too unselective to offer a viable solution for complex synthetic problems. Radical intermediates often have a number of different reaction pathways available to them that are all associated with insubstantial reaction barriers so that reaction outcomes can be controlled by proximity and dynamics. Cage effects consist of the effect of the surrounding medium, such as the solvent or the enzyme pocket, on the movement of radical intermediates and the medium’s resulting influence over reaction outcomes and selectivity. Cage effects substantially affect the outcome of all transformations in condensed phases, which feature the intermediacy of radical pairs, and a suitable choice of the cage should thus constitute a key optimization parameter for radical reactions. This Perspective provides an overview of key aspects of the cage effect that can be of importance in synthetic chemistry and highlights its role in a number of recently reported transformations that forge C–X bonds via the intermediacy of radicals.

## 1 Introduction

In a seminal paper published in 1934, Franck and Rabinowitch proposed an explanation for the substantially lower quantum yields observed for the photolysis of diatomic molecules such as I_2_ in solution compared to the gas phase: the cage effect.^1^ Iodine in solution is surrounded by solvent molecules, and when it undergoes photolysis to form two iodine atoms, the diffusion of the iodine atoms away from one another is hindered by solvent molecules. For the first ∼10^–11^ s after photolysis, the iodine atoms must remain at a distance that is close to the bonding distance of iodine because the surrounding solvent molecules have not yet been sufficiently rearranged to permit the iodine atoms to separate from one another. Due to the enforced proximity of the two radicals in the solvent cage over a period of time corresponding to >10^3^ molecular vibrations, the two radicals are likely to react with one another to reform I_2_.^2^ However, the cage effect is not just a solvent effect; it also influences the course of reactions in enzyme pockets, on heterogeneous surfaces, in the pores of zeolites, and upon encapsulation in micelles. Considering that almost all reactions in synthetic chemistry are carried out in condensed phases, cage effects influence the vast majority of practically relevant radical reactions.

The phenomenon underlying the cage effect is that transformations of radical pairs are controlled principally by proximity and dynamics rather than reaction barriers because radical intermediates tend to have many different reaction pathways available to them, all of which are associated with negligible activation barriers. While selectivity control for reactions between radicals is subject to dynamic control rather than energetic control, the degree of selectivity that can be achieved is nonetheless extremely high. A tightly fitting cage provided by a protein pocket, for example, can ensure that unreactive C–H bonds in complex molecules are functionalized with near perfect regio- and stereoselectivity.^3^ Enzymes such as the various members of the P450 class have inspired chemists for decades with a desire to both understand and mimic their ability to selectively hydroxylate C–H bonds in alkanes, epoxidize olefins, and dealkylate tertiary amines. It is remarkable that highly reactive species such as alkyl radicals can be generated in a biological environment, and undergo selective in-cage C–X bond formations rather than causing havoc.^4^ Effective synthetic mimics of P450 enzymes need not only provide a suitable active site but also fulfill the function played by a well-fitting enzyme pocket, which exerts control over the movement of radical intermediates.

While an increasing number of new transformations reported in organic chemistry feature radical intermediates, the role played by cage effects is rarely discussed explicitly.^5−7^ Strategic use of cage effects to direct the selectivity of transformations is thus a potentially valuable tool, especially in C–H functionalization reactions, where the lack of polarity in the C–H bond favors homolytic over heterolytic bond cleavage as a bond activation modality. Even if it is not intentionally used to direct the course of reactions, the cage effect must be considered in a variety of contexts, such as the determination of bond dissociation values from bond homolysis processes in solution or the interpretation of radical clock experiments. This perspective illustrates various ways in which cage effects can affect the efficiency, regioselectivity, and stereoselectivity of transformations involving radical pairs and illustrates these points with examples of recently reported transformations in which cage effects play a notable role.

Historically, the impetus for the study of cage effects was provided both by their relevance to crucial biological transformations involving P450 enzymes and coenzyme B-12, as well as by their importance in free radical polymerization, which is widely applied in the production of plastics.^8−11^ Radical polymerization reactions commonly commence when an initiator molecule, such as AIBN undergoes thermal fragmentation into a geminate radical pair, and the radicals escape the solvent cage and react with a closed shell species to form the chain carrying radical. However, about 40% of the activator used is usually wasted because the radical pair that is formed does not escape the solvent cage but instead undergoes recombination to form a product that can no longer undergo thermal homolysis under the reaction conditions.^12^ In addition to initiation, the termination events of polymerization reactions involve reactions between pairs of radicals to form a closed shell species. Both the average chain length and the distribution in chain lengths that is obtained crucially affect the properties of the polymer product so that the control of radical-closed shell species and radical–radical reactions are of substantial economic importance.

## 2 Fundamentals of Radical Pairs

**Figure 1:**
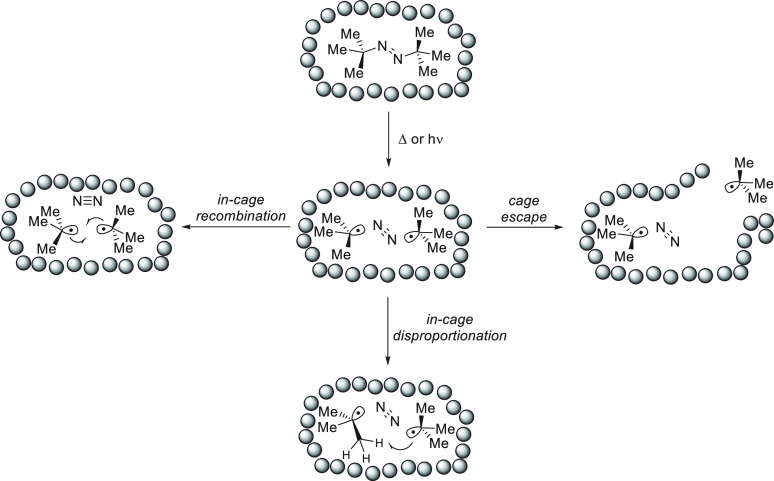
Generation of a radical pair in a solvent cage (solvent molecules are represented by gray spheres) which can undergo recombination, disproportionation, or cage escape.

Given that radical–radical recombination reactions have low or even no activation energy, recombination reactions generally take place whenever two radicals collide in a suitable mutual orientation. The geometric constraints on productive reaction encounters depend on the structures of radicals. Carbon-based radicals generally favor a planar or nearly planar structure unless the radical center is part of a ring system that prevents planarization. Methyl radical, for example, is perfectly planar, with the unpaired electron residing in a p orbital, so that orbital overlap with a second methyl radical to bring about C–C bond formation can easily be achieved.^13,14^ The CF_3_ radical, on the other hand, has a pyramidal structure that prevents unfavorable interactions between the SOMO and the fluorine lone pairs and permits hyperconjugation between the SOMO and σ* C–F bonds.^15^ The *tert*-butyl radical has an estimated degree of pyramidalization that corresponds to 40% of that present in a perfect tetrahedron, and an inversion barrier of ∼2.6 kcal·mol^–1^.^13,16−19^ Pairs of methyl radicals react via recombination at nearly every collision in the gas phase, but for radicals with a more complex structure, disproportionation may compete substantially with recombination as a pathway to closed shell products (Figure 1).

The presence of a cage surrounding a pair of radicals implies that two sets of behaviors of the radicals need to be considered: those taking place inside the cage and those taking place after a radical has escaped the cage. Experimentally, the use of scavengers is common to differentiate between transformations that take place within and outside of a solvent cage. Scavengers are chemicals that react with the radical in question in a rapid and irreversible manner once it has escaped the solvent cage in which it was formed.^2,20,21^ Random recombination of free radicals in solution is kinetically outcompeted by a suitably high concentration of scavenger, but the fraction of product that is generated by radical–radical recombination within the solvent cage remains unaffected until the scavenger concentration becomes so high that it constitutes a cosolvent. Scavengers present in cosolvent quantities are statistically likely to form part of the solvent cage surrounding the caged radical pair, which enables the scavenger to react even with a caged radical. Experimental evidence can be supported with theoretical considerations regarding the likelihood of cage escape for a particular type of radical pair.^2,22,23^ The Noyes equation permits the calculation of the probability of cage escape as a function of the initial separation between two radicals, their mass, radius and translational energy as well as the viscosity of the medium.^2^

### 2.1 The Importance of Radical Spin State

**Figure 2:**
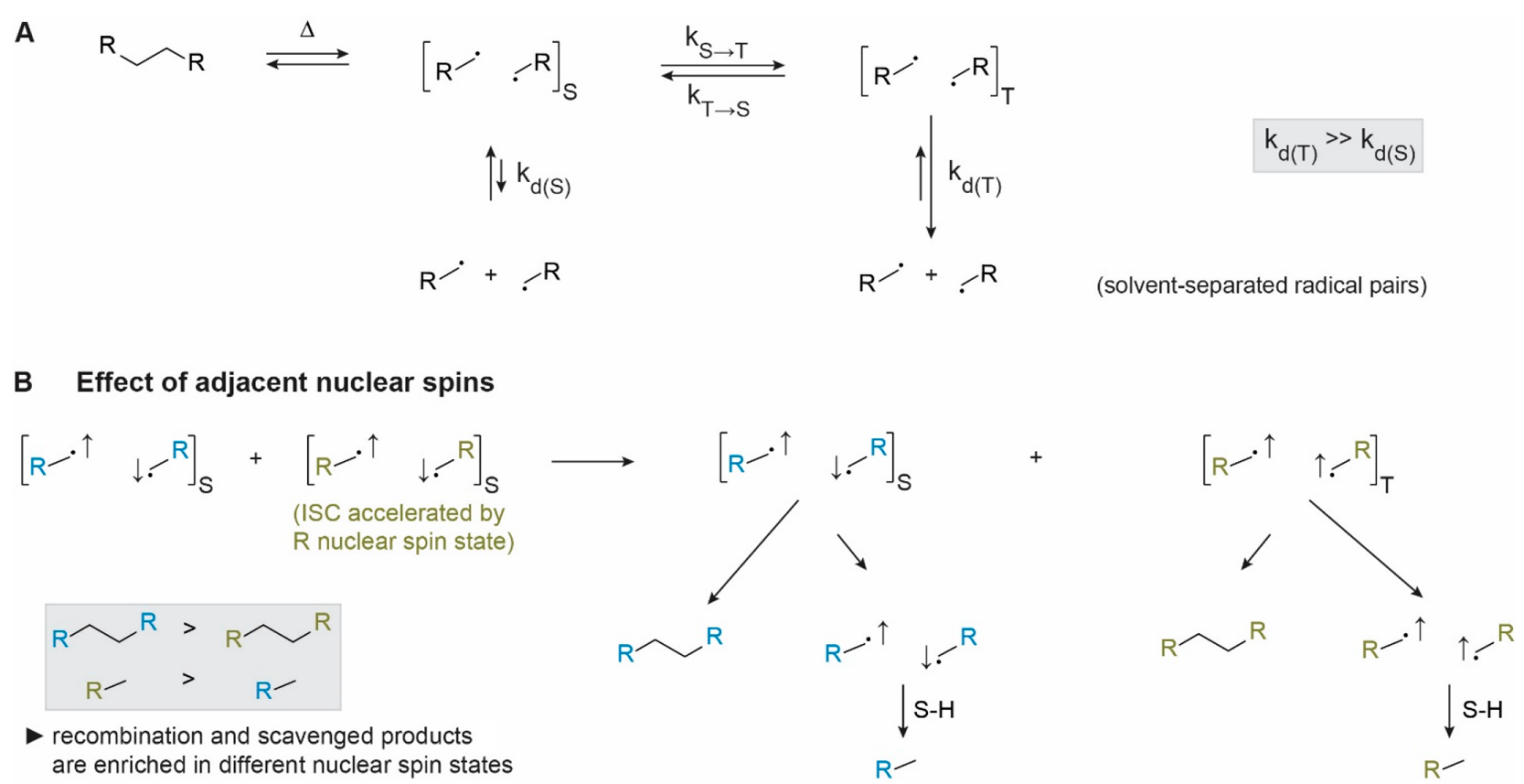
(A) The probability of cage escape is higher for a radical pair in a triplet state because ISC needs to occur before radical–radical recombination can take place. (B) Adjacent nuclear spins influence the rate of intersystem crossing, which in turn affects the fraction of radicals undergoing cage escape (square brackets represent the solvent cage, S and T indicate whether the radical pair is in a singlet or triplet state, and blue and green colors represent different nuclear spin states of the substituent R).

A system comprising two carbon-centered radicals in a triplet state can only engage in C–C bond formation once intersystem crossing (ISC) has taken place to convert the triplet radical pair to a singlet radical pair (Figure 2A). If two radicals with “up” spin that make up a triplet radical pair approach each other, bond formation is not possible because both “up” spins would need to populate the same molecular orbital, which contravenes the Pauli exclusion principle. Due to the strong spin dependence of radical recombination reactions, the fate of radicals is substantially affected by whether the process that gives rise to the formation of the radical pair favors the formation of either singlet or triplet radial pairs. Since thermal bond cleavage commonly leads to singlet pairs and photochemical fragmentation can generate triplet radical pairs, the mode of bond cleavage employed can tune the reactivity of the resulting radical pair. Furthermore, because intersystem crossing interconverts singlet and triplet radical pairs that show different reactivity, any factor that changes the rate of intersystem crossing can result in reactivity differences that vary depending on the degree to which the radical pairs are caged.

An illuminating example of the importance of radical pair spin states was provided by Sakurai and co-workers, who compared the outcome of direct and triplet-sensitized photolysis of *N*-(1-naphtholyl)-*O*-(*p*-toluolyl)-*N*-phenylhydroxylamine.^24^ The molecule in question features an unusually large energy gap between the S_1_ and T_1_ level of 31 kcal·mol^–1^, which renders ISC very inefficient. In the case of nonsensitized photolysis, therefore, homolysis of the weak N–O bond takes place exclusively from the singlet excited state. 1,3- and 1,5-aroyloxyl-migrated in-cage products are obtained along with products from radical cage escape followed by H atom abstraction. The product ratios vary with solvent polarity and can be shifted toward the sole formation of in-cage rearrangement products by the use of a 0.1 M solution of hexadecyl trimethylammonium chloride (HTAC), which leads to the formation of micelles. Triplet-sensitized photolysis, on the other hand, leads to the exclusive formation of products derived from H atom abstraction because intersystem crossing of the caged triplet radical pair is slow and unable to compete with radical cage escape.^24^

The effect of the spin state of a radical pair on reaction outcomes and efficiencies thus depends on the history of the caged pair, and on the available mechanisms for preserving, interchanging and losing spin correlation. In nonviscous (<10 cP) homogeneous solutions at ambient temperature, the lifetimes of solvent cages are on the order of ∼10^–10^ s, the time scale for formation of cages by secondary geminate pairs is on the order of ∼10^–8^ s, and the time scale for formation of caged radical pairs from random encounters between radicals is ∼10^–6^ s.^25−27^ In the absence of heavy atoms that introduce substantial spin–orbit coupling, intersystem crossing between a singlet and a triplet spin state of a radical pair typically takes ∼10^–8^ s, and electronic spin relaxation (by random mechanisms) takes about 10^–6^ s.^1,7,28^ Spin correlation is thus preserved for primary geminate radical pairs, and it may be preserved or interchanged but not lost for secondary radical pairs. An accessible an informative overview of the varying time scales on which numerous chemical and physical processes proceed is provided in the Nobel Prize lecture of Ahmed Zewail.^29^

Unlike the pronounced effect of electron spins, nuclear spins generally have a negligible influence on chemical reactivity. Nuclear-electronic hyperfine coupling, which describes the interaction between the magnetic moments of nuclei and the magnetic moments of electrons, however, provides a vehicle for nuclear spins to influence the reactions of radical pairs (Figure 2A).^30^ An influence of the nuclear spin is possible if the chemical reaction under consideration is not too fast (spin angular momenta will be conserved) or too slow (spin angular momenta will be randomly dispersed). A relaxed cage is a suitable environment for nuclear spins to exert an influence over radical reactivity because radical pairs that are generated within such a cage experience a certain degree of diffusional and rotational freedom, but eventual re-encounter between the radicals remains likely. Consequently, the radicals spend enough time sufficiently far from one another that the energetic difference between the singlet and triplet states is small and comparable in magnitude to the hyperfine energies.^30,31^ The nuclear spin can thus promote singlet–triplet interconversion, which changes the likelihood of bond formation between the radicals when they encounter each other again (Figure 2B).

The pronounced influence of the spin state of a radical pair on the likelihood of recombination, in combination with the ability of nuclear spins to change the rate of intersystem crossing forms the foundation of a powerful technique for the detection of radical pair intermediates: chemically induced dynamic nuclear polarization (CIDNP).^32,33^ CIDNP is an NMR-based technique that detects the polarization of molecules that are formed via the downstream reactions of radical pairs. The molecules observed via CIDNP may be identical to (one of the) starting materials so that no net reaction has taken place because the molecule in question underwent fragmentation and radical–radical recombination.^34^ CIDNP constitutes an experimental alternative to a double label experiment or the observation of racemization of a chiral, enantiomerically enriched starting material for the detection of radical intermediates. CIDNP relies on the fact that the products of radical reactions can exhibit nuclear spin state distributions that strongly differ from the thermally equilibrated Boltzmann distributions, which can be detected in an NMR spectrum in the form of highly enhanced absorption or emission signals. The thousand-fold amplification of signal intensity commonly observed in CIDNP relative to routine NMR analysis makes it feasible to detect trace products, short-lived intermediates, or to measure spectra of low abundance nuclei.^35^ A practical advantage of CIDNP is that unlabeled racemic precursors can be utilized to verify the presence of radical intermediates. However, the NMR spectrum of the polarized product has to be collected before the NMR signal has decayed.^36^ CIDNP can thus be measured if the radical reaction can be carried out inside the NMR spectrometer itself, or using a flow NMR setup, which is particularly useful if the radical pairs are generated photochemically.^36−38^ Whether a transformation which features the intermediacy of radical pairs is carried out directly inside an NMR spectrometer can influence reaction outcomes and CIDNP effects, however, because the strong external magnetic field furnished by the NMR spectrometer affects the rate of intersystem crossing, which in turn influences the rate of radical–radical recombination.^36^

The observation of a CIDNP signal proves the existence of a radical path leading to a given product but does not rule out an independent ionic or concerted pathway for its formation. In the reactions of alkyllithium reagents with alkyl bromides or iodides in hydrocarbon solvents, for example, reasonable ionic (S_N_2 or E2) and radical mechanisms can be proposed to account for the observed coupling and disproportionation products.^35^ CIDNP and ESR studies unequivocally support the formation of a singlet pair of alkyl radials, but these experiments cannot rule out that an ionic and a radical mechanism both contribute to the formation of the observed products.^39−41^ For halogen-metal exchange with organolithium reagents in ethereal solvents, which are understood to proceed via nonradical pathways, no CIDNP spectra are observed.

### 2.2 Effect of Cage Identity

The cage enclosing radical pairs in condensed phases can either be largely static, such as that provided by the pore of a zeolite, or exhibit its own dynamics, as is the case for cages provided by solvent molecules. For radical pairs in a homogeneous solution, the extent to which the radicals are constrained to remain and react within the cage depends on the ease with which solvent molecules rearrange, which is quantified on the macroscopic scale by the solvent’s viscosity. For nonviscous solvents, cage effects are generally small.^19^ For example, only 4% of radicals generated via photolysis of (*S*)-α-methyldeoxybenzoin in benzene solution underwent in-cage recombination.^42^ In the case of highly reactive radicals, for example, methyl radicals generated in the photolysis of azomethane, substantial cage effects can be observed even in nonviscous solvents. For example, azomethane photolysis in hexane at 20 °C generated 73% hexane and 54% methane (two molecules of methane can be generated from each molecule of azomethane).^43^ Kodema carried out detailed studies on the effect of solvent polarity as well as reaction temperature on caging efficiency, and demonstrated a pronounced increase in the cage efficiency as the temperature is lowered, followed by an additional increase as the solution freezes.^44^ Probing the effect of solvent viscosity on reaction outcomes has long been employed as a means to study the contribution of in-cage radical–radical combination or to favor either in-cage transformations or cage escape.^45^ In 1974, for example, König and Owens reported that both the yield and the optical purity of the ether radical recombination product in the thermal decomposition of (*S*)-*N*-nitroso-*N*-(α-methyl)-butanoyl-*O*-*tert*-butylhydroxylamine is dependent on the viscosity of the solvent, exhibiting an increase in the enantiomeric excess from 1.3% in pentane, 2.3% in dodecane, and 9% in nujol.^20^ The use of scavengers highlighted that very little product is formed via random radical–radical recombination following the cage escape. In 2016, Tyler et al. showed however that the bulk viscosity of the solvent is an inadequate descriptor for the solvent behavior on the time scale of cage rearrangement.^46^ Observing that the cage effect is a very localized phenomenon, they showed that a solvent’s microviscosity of a solvent shows a more consistent correlation with its caging ability.^47^ Notably, the microviscosity of a solvent can be determined in a straightforward fashion via diffusion ordered spectroscopy (DOSY) NMR studies with a stable analogue of the radical species of interest.^46^

Compared to an isotropic medium such as an organic solvent, the presence of phase boundaries decrease the mean free path of a radical pair and thus lead to enhanced cage effects.^48^ For example, the initiator di-*tert*-butylhyponitrite demonstrates an efficiency of 0.66 for the formation of free radicals in chlorobenzene, while the efficiency sinks to 0.3 for a 0.5 M aqueous solution of sodium dodecyl sulfate (SDS), due to the confinement of di-*tert*-butylhyponitrite inside the micelles formed by SDS. In the presence of dipalmitoyl-phosphatidylcholine (DPPC) liposomes, the efficiency of radical generation from di-*tert*-butylhyponitrite sinks even further to 0.09.^19^ In addition to the intrinsic interest in the enhancement of cage effects due to movement restriction, the importance of free radical autoxidation in biological membranes provides an impetus to deepen our understanding of the radical behavior in micelles and bilayers.

When the cage effect for photolysis of a ketone was investigated in the presence of different detergents that all form micelles in aqueous solution, it was found that the length of the alkyl chain in the sodium alkyl sulfate detergent strongly correlates with the extent of the cage effect. A longer hydrophobic tail on the surfactant gives rise to a thicker wall for the micelle through which the radical has to pass in order to escape from its cage, so that the cage efficiency could be varied from 4% for a detergent chain length of 6 to a cage efficiency of 50% for a chain length of 14.^25^ The efficiency of acyl radical capture for a photo-Fries rearrangement under flow conditions could also be substantially enhanced via the addition of surfactants that led to the confinement of radical intermediates in micelles.^49^

**Figure 3:**
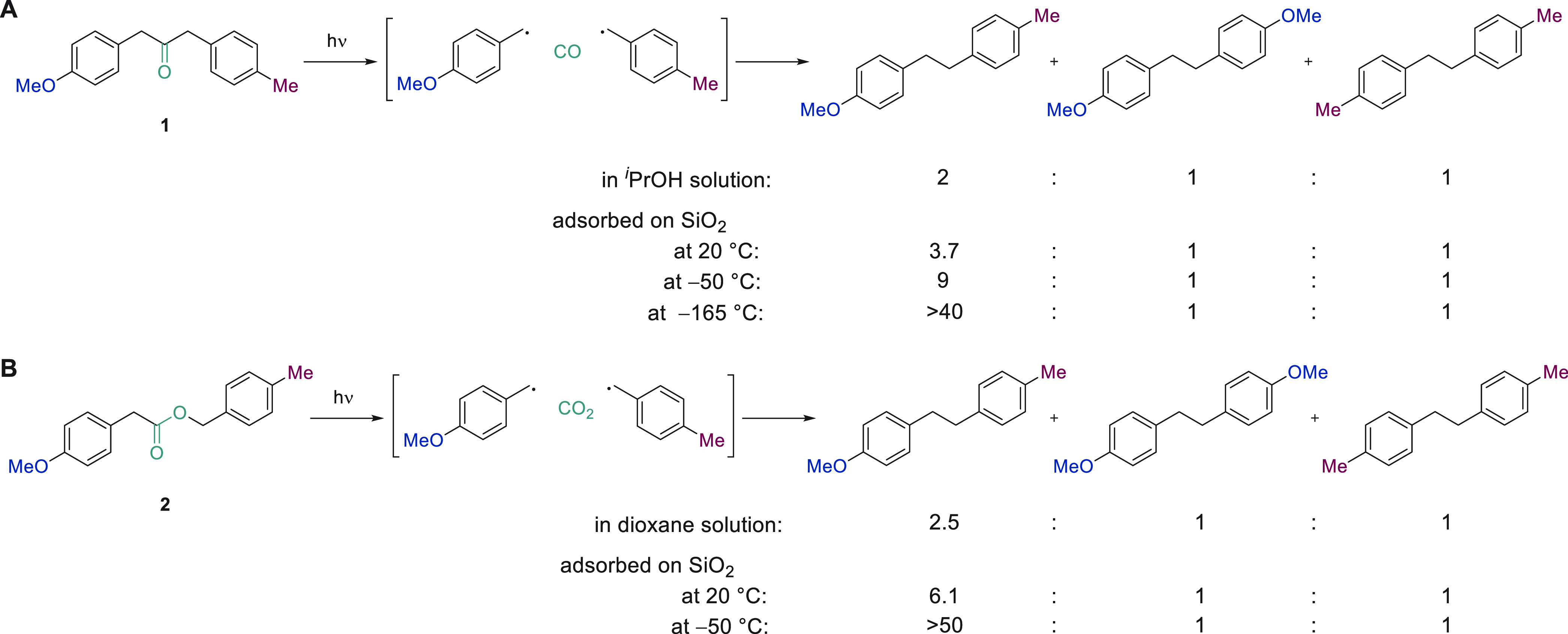
Effect of silica adsorption of the precursor and temperature on the product distribution obtained in photo-Fries rearrangements.^52^

In addition to compartmentalization in soft matter such as within polymers, micelles, or liquid crystals, radical pairs can also experience increased confinement in hard matter phases such heterogeneous surfaces, or within porous materials and thus demonstrate notable cage effects.^50^ While the photo-Fries rearrangement of an unsymmetrical ketone (Figure 3A) or ester (Figure 3B) in isotropic solution gave rise to a close to statistical mixture of all possible products, adsorption of either precursor on silica favored the coupling of radicals generated in close proximity from one another.^51,52^ A decrease in the reaction temperature further lowered the mobility of the adsorbed species on the silica surface and ensured a higher degree of selectivity for the formation of the unsymmetrical product.

**Figure 4:**
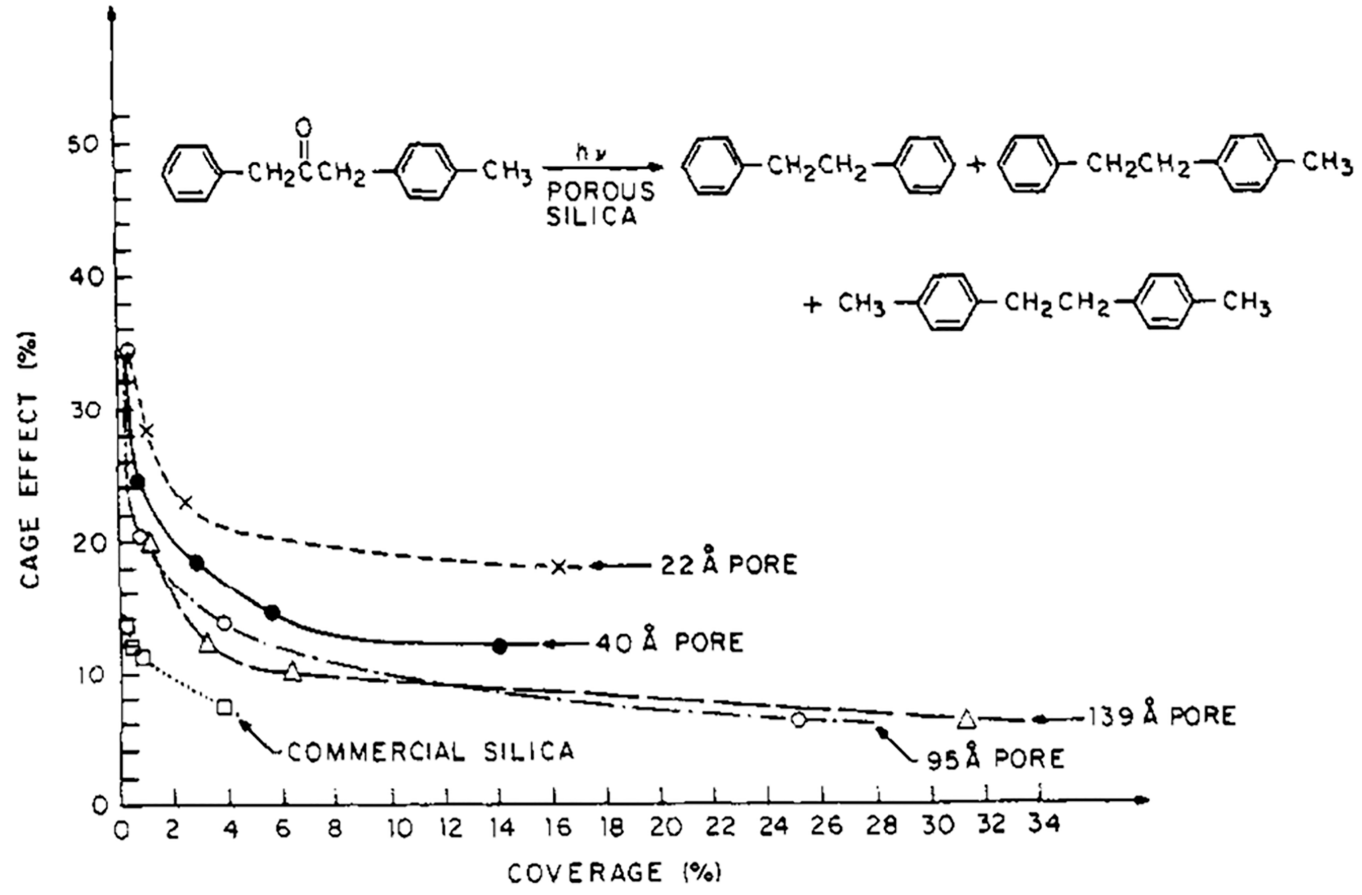
Cage effect for the photolysis of a ketone as a function of silica pore size.^63^ Adapted from ref (63). Copyright 1984 American Chemical Society.

A separate study of the photo-Fries rearrangement of ketones adsorbed on silica demonstrated a notable dependence of the extent of the cage effect on the silica pore size (Figure 4). For commercial silica, which was also used in the work described in Figure 3, only a very small cage effect was observed at room temperature due to the presence of pores of various sizes, many of which are >100 nm. For a low degree of substrate coverage on the support surface and a pore size of 22 Å, however, cage effects exceeding 30% could be reached at room temperature. Even more pronounced cage effects are accessible in zeolites, which contain pores in the range of 10 Å.^53^ Crucially, the atomically precise structure of zeolite materials ensures that the extent of the cage effect can be tuned with precision since materials can be selected for which all pores have the same shape, diameter, and an almost identical chemical composition of all inner surfaces.

In a detailed study, Turro and co-workers examined the fate of geminate pairs of carbon-centered radicals inside zeolites for which strongly acidic sites were removed via cation exchange of protons with alkali metal ions.^53^ A triplet radical pair was photochemically generated so that geminate recombination could only occur after intersystem crossing and relative rotation, diffusion, or decarbonylation of the initially formed radical can take place before the radical pair undergoes geminate recombination. The constriction of the zeolite matrix was able to reverse the selectivity of the radical–radical reaction between methylbenzyl radicals from 95% combination, as was observed in solution, to almost exclusive disproportionation. The steric hindrance encountered by two radicals located at the intersection of the channels in an MFI zeolite disfavors combination relative to disproportionation, which is less easily inhibited by steric constraints because a larger number of relative alignments of the two species lead to a productive encounter. Supramolecular constriction can also lead to radical persistence: While the majority of diphenylmethyl radicals generated by photolysis of tetraphenylacetone adsorbed on LZ-105 zeolites were transformed into closed shell species on the millisecond time scale, 2–7% of the radicals remained stable over the course of weeks.^54,55^ Upon photolysis, the ketone precursor undergoes α-elimination to yield a diphenylacetyl radical, which undergoes decarbonylation within 10 ns. In homogeneous solution, diphenylmethyl radical undergoes nearly diffusion controlled radical–radical coupling to furnish a quantitative yield of 1,1,2,2-tetraphenylethane. By matching the size of the radical intermediate to the channel dimensions of the MFI type zeolite, however, photolysis of the ketone precursor occurs on the zeolite surface, and a small fraction of the radicals thus generated can enter the channels of the zeolite.^56^ The diameter of diphenylmethylradical (∼5.7 Å) renders diffusion along the MFI channels (5.5 Å) challenging, so that radical–radical encounters become very rare and diphenylmethyl radical is rendered kinetically stable.^54^

An often neglected aspect of the solvent cage is the presence of a gas molecule.^57^ The cage effect for radicals pairs that are separated by a third molecule, such as gaseous N_2_ when the radical pair results from the photodecomposition of a diazene, are reduced by the need for the radicals to move “around” N_2_ in order to engage in bond formation (Figure 1). The influence of precursor-derived closed shell species is frequently ignored in the discussion of cage efficiency despite the fact that the diffusion constants for gas molecules released during radical generation are on the same order of magnitude as those for the molecules making up the solvent cage, which is assumed to remain close to static on the time scale of primary radical recombination.^58,59^

### 2.3 Control over Regiochemical Outcomes

**Figure 5:**
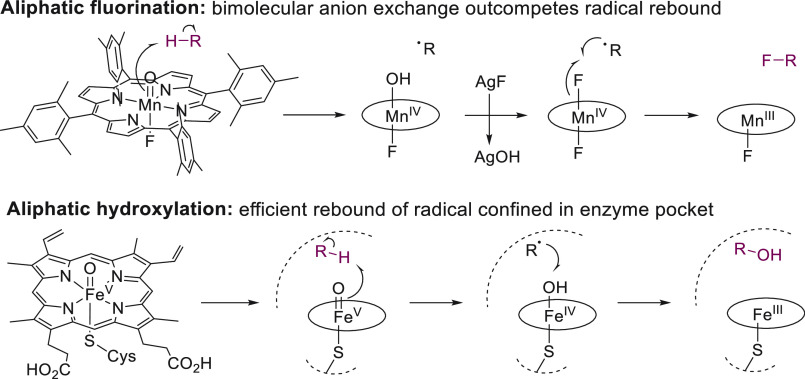
Reaction outcomes of aliphatic fluorination and hydroxylation imply substantial differences in the lifetime of alkyl radical intermediates generated via hydrogen atom abstraction.^60^

A problem that has been studied in great detail in a variety of settings is the control over the selectivity of reactions between hydrocarbons and porphyrin ligated metal-oxo species.^6^ As a central component of the P450 enzyme family, the porphyrin iron oxo active site has been studied for decades both within its enzyme host as well as in the context of biomimicry, in homogeneous solution. Furthermore, a number of transformations for which no biological equivalent exists have also been developed, which make use of radical intermediates generated from high-valent metal oxo catalysts.^3^ A notable example of the latter was the development of manganese-catalyzed aliphatic fluorination with anionic fluoride (Figure 5) reported by Groves and co-workers in 2012.^60^

**Figure 6:**
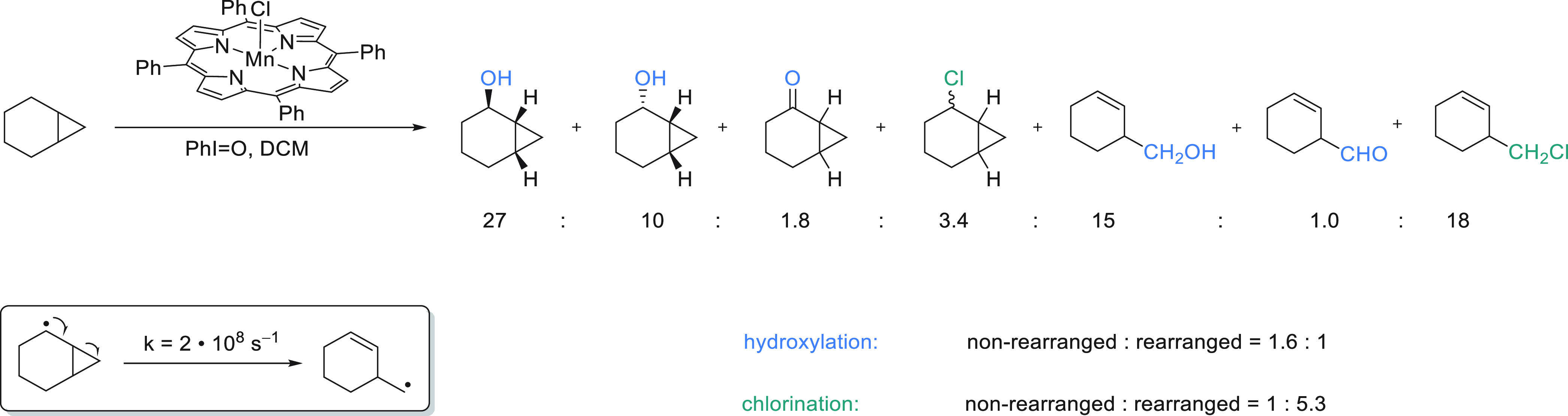
C–H functionalization of a radical clock substrate catalyzed by a porphyrin supported manganese oxo.^61^

Early investigations showed that oxidation of cyclohexane with PhI=O catalyzed by porphyrin Cl–Mn=O in dichloromethane gave rise to a 2.5:1 ratio of the hydroxylated and the chlorinated product in a combined yield of 70%.^61^ When the dichloromethane solvent was replaced with dibromomethane or bromotrichloromethane, cyclohexyl bromide was even formed as the major product.^61^ On the other hand, chlorinated products were also observed when porphyrin Cl–Mn=O catalyzed oxidation was carried out in benzene, where the amount of chlorinated product obtained was consistent with the amount of catalyst present. Consequently, alkyl halide products could be obtained both via cage escape of alkyl radicals followed by halogen atom abstraction from a solvent as well as via in-cage radical rebound reactions with porphyrin Cl–Mn–OH. To determine whether hydroxylation and chlorination proceed via in-cage or cage escaped radical intermediates, the radical clock substrate norcarane was subjected to manganese-catalyzed C–H functionalization. When norcarane undergoes hydrogen atom abstraction, the resulting norcaran-2-yl radical undergoes ring opening with a rate constant of k = 2 • 10^8^ s^–1^, which allows one to benchmark the lifetime of radical intermediates formed over the course of the reaction (Figure 6).^62^ Both chlorination and hydroxylation are observed with and without concomitant rearrangement. It is notable, however, that the extent of rearrangement was substantially higher for chlorinated products compared to hydroxylated products. Olefin epoxidation was studied with the same manganese oxo system and cis-stilbene was found to yield a 1.6:1 mixture of the *trans*- and the *cis*-epoxide, which the authors attribute to Mn(V)=O adding to the stilbene double bond to yield a freely rotating free-radical intermediate that enjoys a substantial lifetime before undergoing radical rebound.^61^

**Figure 7:**
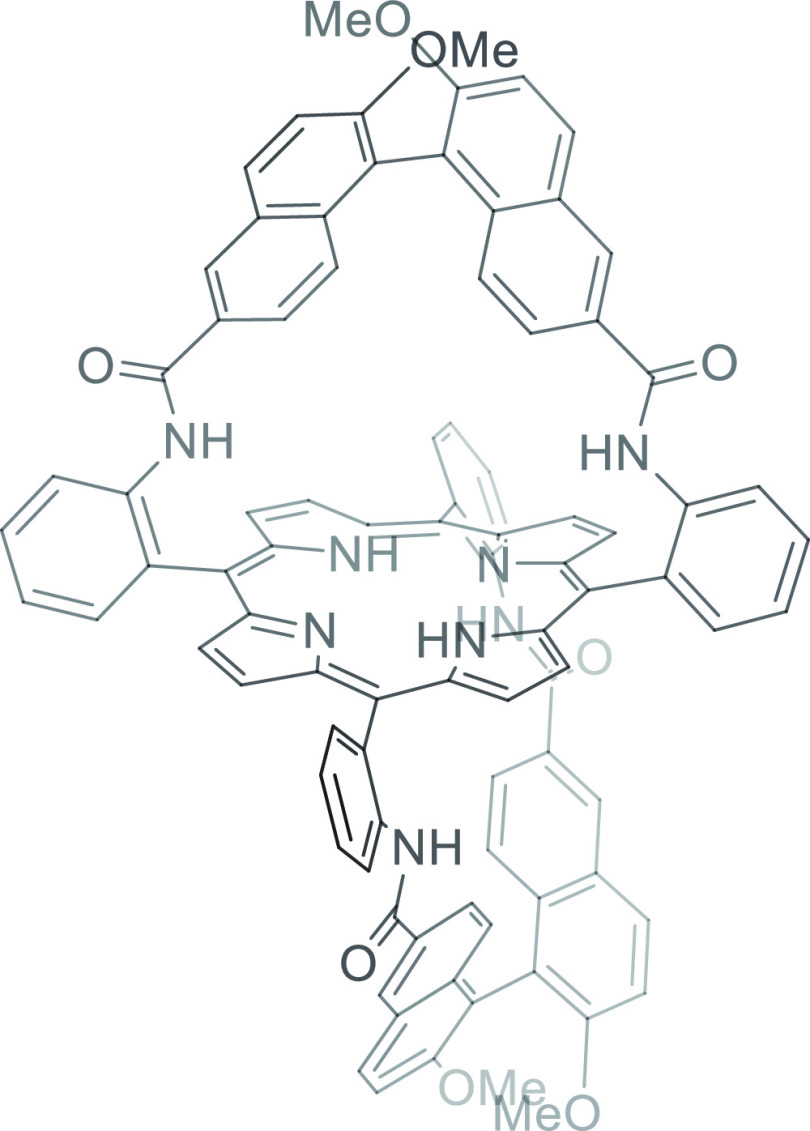
Basket porphyrin ligand used in Mn=O and Fe=O catalyzed aliphatic oxidation by Groves and co-workers.

A study involving Fe=O and Mn=O ligated by basket porphyrins (Figure 7) by Groves and co-workers shed some light on the origin of the ketone side products commonly obtained in aliphatic oxidation, which had also been observed in the study described in Figure 6. Ketone or aldehyde formation did not arise from overoxidation of the primary alcohol product, because direct subjection of the alcohol to Fe=O or Mn=O catalyzed oxidation did not give rise to ketone formation.^64^ Instead the ketone is likely formed by reaction between the alkyl radical intermediate with either oxygen or the stoichiometric oxidant, iodosylbenzene. Based on this assignment, the desired alcohol product is formed via radical rebound, which occurs within the cage, while the ketone side product arises from cage escape.^65^ In the case of the basket porphyrin studied, the Fe=O catalyst consistently furnished higher alcohol to ketone ratios, which is in line with the longer lifetime of radical intermediates for manganese versus iron porphyrins.^61,65^ Notably, the “handle” of the basket porphyrin ligand provides a chiral pocket so that the enantiomeric excess of the product can function as a reporter of the extent of interaction between the substrate and the chiral pocket provided by the basket. Larger substrates for which a better fit with the basket was expected not only provided higher enantiomeric excess, but also furnished the lowest amounts of ketone side product.^64^

**Figure 8:**
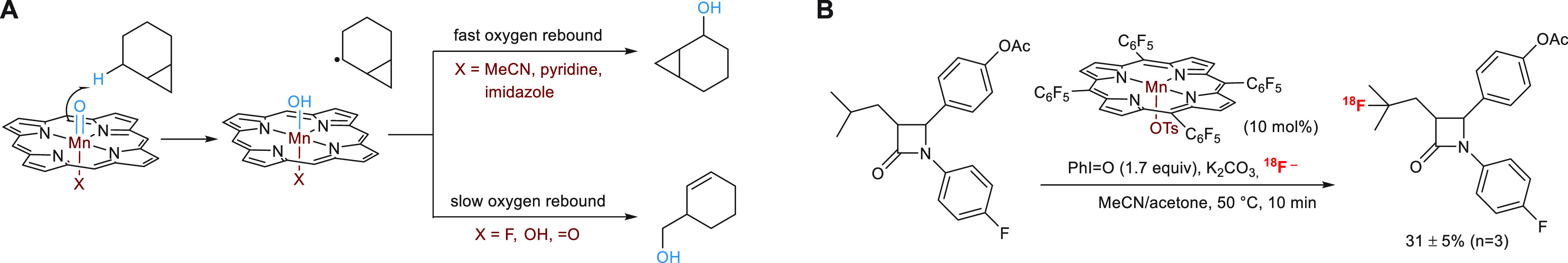
Capitalizing on the effect of the trans-axial ligand on the rate of oxygen rebound (A). Groves and co-workers were able to develop Mn=O catalyzed C–H fluorination with ^19^F and ^18^F (B).^6,60,70^

While the slower radical rebound reaction between carbon centered radicals and Mn–OH centers compared to Fe–OH centers is a disadvantage for the development of selective hydroxylation reactions, it can be advantageous in order to promote C–X (X ≠ O) bond formation. Catalytic systems that are capable of abstracting strong C–H bonds under mild conditions are scarce, so selecting reaction conditions under which the radical intermediate formed via hydrogen atom abstraction by metal oxo catalysts can undergo efficient cage escape is an attractive alternative to the development of different HAT catalysts. Formation of C–X bonds is possible if cage escape of the alkyl radical increases its lifetime sufficiently for ligand exchange at the metal catalyst to become kinetically competitive with radical rebound (Figure 5). In 2002, the use of high-valent Mn porphyrins was investigated for the transfer of X = Cl, Br, I, or N_3_ to alkyl radicals.^66^ While substantial amounts of alcohol were formed alongside the C–X product, two decades later, the same conceptual approach was used with great success to achieve manganese-catalyzed aliphatic fluorination, where alcohol side product formation could be restricted to 15–20% while fluorinated products were obtained in around 50% yield.^60,67^ A fundamental insight that laid the groundwork for the discovery of efficient C–H fluorination with nucleophilic fluoride is the effect of the trans-axial ligand bound to Mn in L-Mn^V^=O on the rate of radical rebound: During mechanistic studies on a C–H bond chlorination reaction they had developed earlier,^68^ Groves and co-workers noticed that axial fluoride ligands have the ability to substantially slow down oxygen rebound of alkyl radicals (Figure 8).^69^ Importantly, a combination of TBAF and AgF was required for manganese catalyzed fluorination because exclusive use of AgF as the fluoride source furnished fluorinated and oxygenated products in an ∼1:2 ratio.^60^ Groves and co-workers carried out manganese catalyzed fluorination of norcarane, which gave rise to a 2:1 ratio of cyclopropylcarbinyl and homoallyl fluorides. Based on a rate constant of *k* = 2 × 10^8^ s^–1^ for the ring opening of 2-norcanyl radical, the lifetime of the radical intermediate was determined to be on the order of 2.5 ns.

Considering that ligand exchange at the manganese center needs to proceed at competitive rates with radical recombination, it is particularly surprising that Mn porphyrin catalyzed fluorination can be used for labeling with ^18^F.^70^^18^F is a radioactive isotope of fluoride with a half-life of 109 min, which is used for the preparation of probe molecules for positron emission tomography (PET) imaging. Due to safety, cost, as well as practical considerations, ^18^F is only present in picomolar to nanomolar concentrations during no-carrier added fluorination reactions.^71,72^ Interestingly, the bimolecular ligand exchange step at the manganese center still proceeded with sufficiently fast rates to permit successful trapping of cage escaped alkyl radicals with manganese centers carrying the radionucleide.^73^

**Figure 9:**
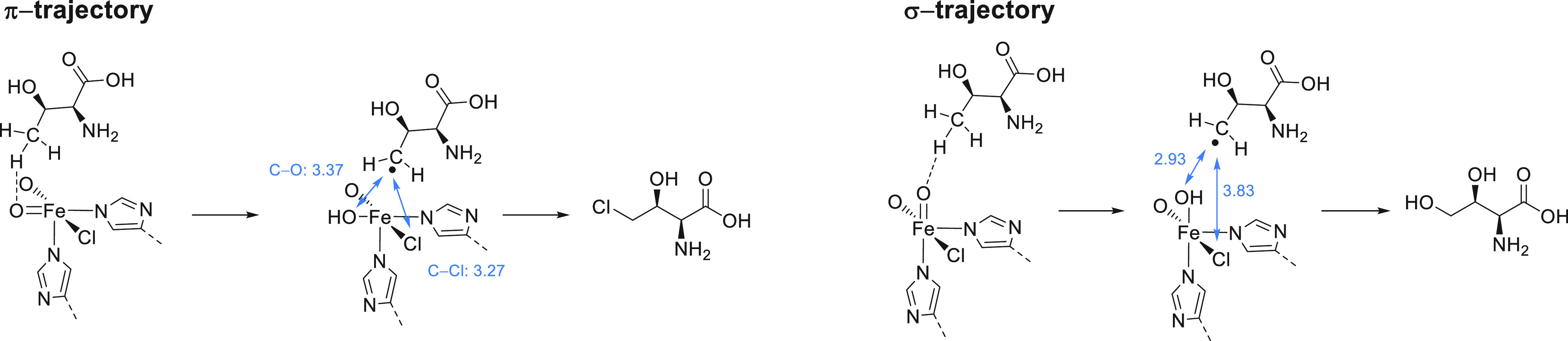
Depiction of the two distinct trajectories is based on calculated pathways for C–H abstraction via either a σ- or a π-trajectory for the native substrate undergoing HAT with SyrB2.^74^

Analogous to the synthetic Mn oxo porphyrin systems, both the C–O and the C-halide containing products can be generated via radical rebound reactions for the nonheme halogenase enzyme SyrB2.^74^ Chlorination and hydroxylation are in-cage reactions, and the mode of the HAT transfer from the substrate to Fe=O positions the substrate so as to favor either chlorination or hydroxylation. The native substrate of the α-ketoglutarate-dependent enzyme SyrB2 is l-threonine, which undergoes enzyme catalyzed methyl group halogenation. While most members of the NHFe enzyme family to which SyrB2 belongs feature an active site in which the iron center is surrounded by a facial triad consisting of two histidines and one carboxylate ligand, SyrB2 contains an active site in which iron is ligated by two histidines and one chloride. If the substrate approaches the Fe=O moiety via a π-trajectory (Figure 9), the resulting alkyl radical undergoes chlorination in preference to hydroxylation, whereas approach via a σ-trajectory leads to preferential hydroxylation. The computed barrier for the π-trajectory is lower than that for the σ-trajectory for the native substrate l-threonine (Figure 9), which is consistent with the experimentally observed preference for chlorination. The difference between the two approach pathways for HAT is that when the radical is formed, it is closer to either the oxygen atom of Fe–OH (σ-trajectory) or the chlorine atom of Fe–Cl (π-trajectory) (blue numbers in Figure 9 show calculated distances to both O and Cl). In-cage radical recombination is facile for either C–O or C–Cl bond formation, so that the reaction selectivity is determined primarily via dynamics. The enzyme’s ability to catalyze both hydroxylation and chlorination was shown with the non-native substrate l-norvaline, for which hydroxylation via a σ-trajectory was the preferred pathway, and hydroxylation was observed.

Maiti and co-workers developed a strategy to favor halogenation over hydroxylation for a synthetic Fe oxo based system, for which the ligand design was inspired by α-ketoglutarate dependent halogenase enzymes.^75^ In a stoichiometric reaction between *cis*-1,2-dimethylcyclohexane with the iron oxo complex supported by a pentacoordinate nitrogen-based ligand, a mixture of *cis*/*trans*-1,2-dimethylhexanol was obtained in 46% yield. The high degree of epimerization was attributed to the formation of a long-lived radical intermediate that can effectively exit the solvent cage. When substrate oxidation by the iron oxo complex is carried out in the presence of a stoichiometric amount of an Fe–Cl or an Fe–Br complex, C–H halogenated products are obtained even for simple alkanes in 46–97% yield, and hydroxylation could be prevented entirely for many substrates.^75^ In 1993, Que and co-workers already showed that chloride, bromide or azide could be transferred from an iron center supported by a tetra-amine ligand to cyclohexane in the presence of an oxidant in >70% yield.^76^

The calculated radical rebound barriers for nonheme based complexes are generally higher than those of heme based systems, whereas Mn=O systems commonly lead to longer-lived radical intermediates compared to Fe=O based catalysts.^6^ For iron porphyrin based systems, the key high-valent iron oxo intermediate can furthermore adopt either a low spin S = 1/2 or a high spin state S = 3/2, depending on the precise nature of the ligand.^77,78^ Shaik and co-workers showed that while both the doublet and quartet spin states of heme Fe=O undergo hydrogen atom abstraction from C–H with similar energy barriers, the barrier for the subsequent radical rebound step is substantially higher on the quartet energy landscape compared to the low spin doublet.^79^ Differences in the facility of radical rebound were also observed for different spin states of nonheme metal oxo systems of manganese, iron, chromium, iron and ruthenium.^3^

Recently, Houk and co-workers carried out a detailed molecular dynamics (MD) simulation to better understand the hydrogen abstraction and radical rebound steps in iron porphyrin catalyzed C–H bond hydroxylation of ethylbenzene.^80^ On the doublet energy surface, 45% of reactive trajectories led directly to the hydroxylated product when quasi-classical MD was carried out in the gas phase, while 56% led directly to the alcohol with an implicit DCM solvent model. The average time gap between the formation of the O–H and the C–O bond of the alcohol increased from 99 fs in the absence of solvent to 156 fs with a DCM solvent model.

### 2.4 Control over Stereochemical Outcomes

When radical pairs are generated in isotropic fluids such as organic solvents or water, the radicals can tumble freely so that even if they undergo radical–radical recombination within the solvent cage in high efficiency, the stereochemical information present in the precursor is usually lost. The prevalent use of isotropic media in which tumbling is rapid, has led to a widespread belief that reactions which proceed via radical intermediates invariably eradicate the stereochemical information from the precursor.^19,81^ Restricting the freedom of movement of radical intermediates constitutes a powerful approach toward controlling the regio- and stereochemical outcome of radical–radical recombination reactions. The topochemical principle states that radical pair reactions in glasses, crystals, and other rigid matrices occur with the least possible motion, so that stereochemical retention is preferred. However, the release of small molecules such as CO_2_ can generate local stresses in the cavity in which radicals undergo recombination so that nonleast-motion products can be observed.^82^

**Figure 10:**
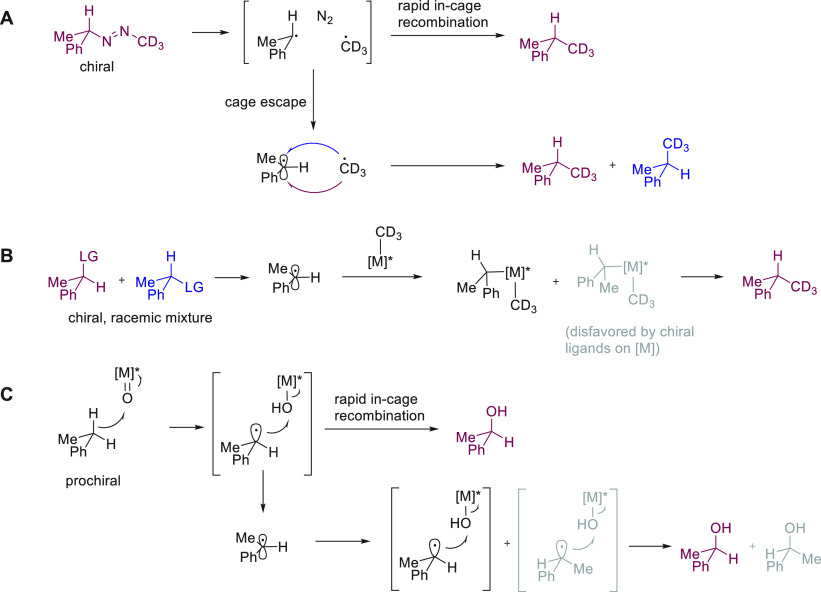
Retention (A) or generation (B, C) of chiral centers during reactions that proceed via a radical mechanism.

Retention of the configuration during transformations with radical intermediates is strategically distinct from reactions in which the initial stereocenter is destroyed and recreated during bond formation through the use of a chiral catalyst. In the latter case, both stereoisomers of the starting material give rise to the same enantiomer of the product as the major product. Full planarization of the radical intermediate and loss of all stereochemical information are thus desirable if the starting material is racemic rather than prochiral because only half of the starting material contains the stereochemical information that is desired for the product. For certain rapid in-cage recombination reactions, on the other hand, stereochemical information encoded in the starting material is preserved, and a chiral catalyst or reagent is not required to obtain an enantioselective radical reaction (Figure 10A). Enantioselective cross-coupling reactions promoted by Ni catalysts exemplify the scenario shown in Figure 10B. To ensure that both enantiomers of the starting material are efficiently converted to the desired enantiomer of the chiral product, the loss of stereochemical information from the reaction precursor is desirable. Based on experiments with radical clocks it was concluded that radicals generated likely escape the solvent cage before recombining with Ni, which carries a chiral ligand sphere that is able to control the absolute stereochemistry of the product formed via reductive elimination.^83,84^ In the scenario depicted in Figure 10C, the in-cage product is formed via two enantiodifferentiating interactions with the metal catalyst, while the product generated from cage escaped radicals only profits from one enantiodifferentiating interaction.^85^ Escaped radicals are free to tumble and can reapproach the catalyst after pyramidal inversion of the radical intermediate (gray structure). Only if the catalyst shows little activity for reaction with the inverted radical intermediate will products from cage escaped radicals also be furnished in high stereoselectivity.

Mechanistic studies with enzymes highlighted early on that transformations known to proceed via the intermediacy of radical intermediates can furnish high degrees of stereoselectivity. In the oxidation of an isotopically chiral methyl group in 1-octane by P450, Caspi and co-workers determined that formation of 1-octanol proceeded with a normal kinetic isotope effect and that the hydroxyl substituent was installed at the position from which the hydrogen atom had been removed.^86^ The Groves group subsequently examined to what extent the stereospecificity is related to the fit between the substrate into the chiral pocket that is provided by the catalyst in their examination of Fe catalyst supported by a basket porphyrin ligand (Figure 7), which serves as a model system for P450 enzymes.^87^ Oxidation of ethylbenzene catalyzed by (basketporphyrin)Fe=O gave rise to a 40% yield of 1-phenylethanol as a 71:29 ratio of the *R* and *S* enantiomers, while (*R*)-(1-deuterioethyl)benzene furnished the *S* alcohol with 16% ee and (*S*)-(1-deuterioethyl)benzene furnished the *R* alcohol with 77% ee. Removal from both the *R* and *S* positions of the hydrocarbon proceeded with a kinetic isotope effect of 6.4, and the catalyst shows a 2-fold preference for removal of the *pro*-R over removal of the *pro*-S hydrogen atom. Notably, while the radical formed by removal of the *pro*-R site is captured with almost complete retention, half of the radical generated from *pro*-S hydrogen abstraction undergoes racemization.^87^ The chiral cavity of the basket porphyrin thus gives varying amounts of support to the radicals derived from the abstraction of one of the two enantiotopic hydrogen atoms. Depending on which hydrogen atom is presented to the Fe=O center, the rest of the molecule can establish a differing degree of stabilizing interactions to the “handle” of the basket porphyrin. Furthermore, the example by Groves et al. demonstrates that elaborate catalysts tend to be required to ensure sufficient control over a radical pair to generate notable amounts of enantiomeric excess for transformations in homogeneous solutions in which the radical intermediates have appreciable lifetimes.

A facile way of restricting the ability of radical intermediates to tumble and rotate is to perform transformations of radical intermediates in the solid state. Seminal studies on radical pairs generated in solids were conducted by Bartlett and McBride who studied the photodecomposition of diazocompounds in frozen cyclohexane glass.^82,88^ Both frozen glasses and crystalline materials constitute extremely tight and unyielding cages, so that the radical pairs formed are effectively prohibited from rotation and tumbling. The minimal movement that is possible within the solid matrix leads to striking stereochemical outcomes: meso-azobis(2-phenyl-3-methyl-2-butane) photolysis in cyclohexane glass led to the exclusive formation of the meso radical combination product while the same reaction in a viscous solvent delivered only a low degree of stereochemical retention.^19^ Movassaghi and co-workers used the stereochemical control achievable in radical recombinations in the solid state to access a late-stage intermediate in the synthesis of (−)-communesin F (see section 3.4).^89^

## 3 Case Studies

### 3.1 Skeletal Editing

**Figure 11:**
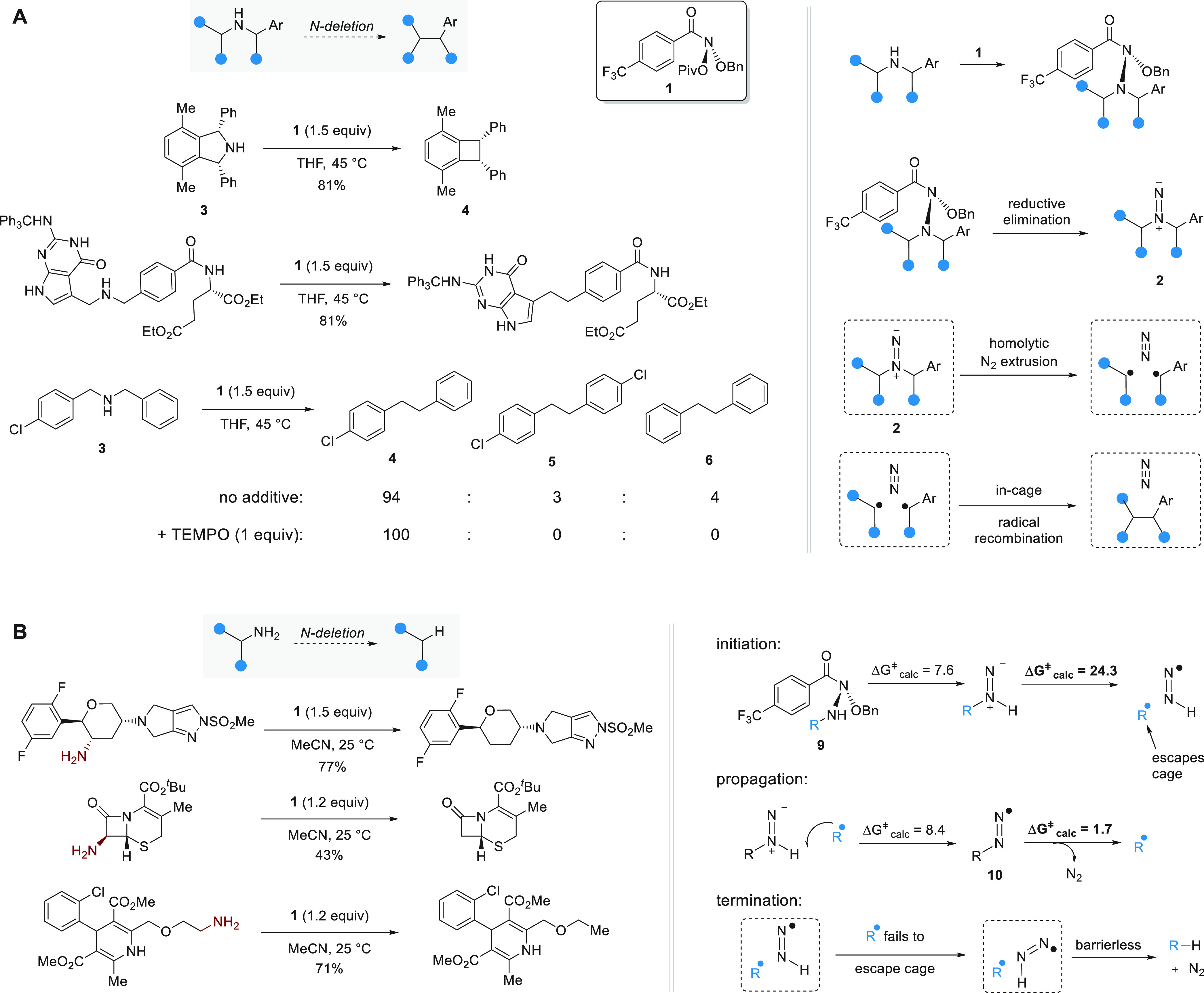
Mechanistic difference for N-deletion from secondary versus primary amines result in a distinct substrate scope for the two transformations developed by Levin and co-workers.^90,96^ Calculated Gibbs energies are given in kcal·mol^–1^, and rectangles indicate solvent cages.

In 2021, Levin and co-workers developed a strategy to “delete” nitrogen atoms from molecules, so that the carbon fragments formerly attached to the nitrogen atom end up connected via a C–C bond.^90−92^ Key to the exploration of the new synthetic disconnection was the development of a suitably functionalized anomeric amide reagent that can be accessed safely and efficiently on multigram scale.^93^ Amides carrying two electron-withdrawing substituents on the amide nitrogen atom (which are termed “anomeric” in analogy to the anomeric position in carbohydrates) are prone to nucleophilic displacement at the nitrogen center.^94,95^ Nucleophilic attack by an amine substrate on anomeric amide reagent **1** followed by reductive elimination yields highly reactive isodiazene **2** (Figure 11A). For secondary amine substrates, the isodiazene intermediate undergoes homolytic C–N bond cleavage to release a pair of carbon centered radicals separated by one molecule of N_2_ inside the THF solvent cage. Efficient radical–radical recombination of the caged pair ensures that two different carbon fragments originally bound to N in the secondary amine substrate are brought together with only 1–10% scrambling. The observation of small amounts of homocoupled **7** and **8** for unsymmetrical amine precursors such as **5** forms part of the evidence that N deletion proceeds via the formation of radical pairs (Figure 11A). Furthermore, addition of radical trapping reagent TEMPO arrests the formation of homocoupled products **7** and **8**, while the yield of desired heterocoupled product **6** was not notably affected. The homocoupling products can thus be attributed to radical intermediates that have undergone cage escape, whereas the dominant reaction pathway, which leads to the desired heterocoupled product **6**, proceeds via rapid in-cage radical–radical recombination. Formation of the caged radical pair relies on thermal cleavage of a C–N bond at 45 °C, so that at least one of the two carbon centers undergoing bond formation needs to carry an aromatic or heteroaromatic substituent in the β position to nitrogen to render N deletion feasible. Given the substantial driving force of C–C bond formation from a radical pair, however, the formation of strained rings such as cyclopropanes or cyclobutanes is possible (Figure 11A). Furthermore, cyclobutane **4** is formed stereospecifically from cis-**3**, which shows that radical recombination, at least for cyclic precursors, is sufficiently fast that stereochemical information from the precursor is transferred to the product.

Follow-up work from Gutierrez and Levin showed that primary amides can also be subjected to N-deletion (Figure 11B).^96^ Unlike the anomeric amide intermediates obtained via the reaction of **1** and a secondary amine, those obtained when **1** reacts with a primary amine (**9**) undergo fragmentation via a radical chain mechanism. A small fraction of **9** undergoes unassisted fragmentation via an energy barrier of Δ*G*^⧧^_calc_ = 24.3 kcal·mol^–1^ (R = Et), and the alkyl radical formed escapes the solvent cage and abstracts a hydrogen atom from another anomeric amide **9**. Consequently, subsequent decomposition occurs via radical intermediate **10**, which can extrude N_2_ even if the R group it carries does not provide substantial stabilization to an adjacent radical center via a calculated energy barrier of Δ*G*^⧧^_calc_ = 1.7 kcal·mol^–1^ for R = Et. The use of a radical clock substrate for which the rate constant for rearrangement is *k* = 4.9 · 10^7^ s^–1^ largely gave rise to rearranged deaminated product, which indicates that the lifetime of the radical species present during deamination are substantially longer than the estimated time required for cage escape (∼10^–10^ s^–1^).

Addition of the stable radical TEMPO furnished high yields of the trapped products alongside little to no deaminated product in contrast to TEMPO trapping carried out for N-deletion from secondary amines, for which high yields of the expected reaction product were still obtained.^90,96^ The divergent outcomes of TEMPO trapping experiments validate that N-deleted products were obtained from cage-escaped radicals for primary amines, whereas secondary amines undergo skeletal editing through an in-cage process. The more extensive substrate scope of nitrogen deletion for primary amines versus secondary amines can be attributed to the reaction proceeding via a radical chain mechanism, for which cage escape of the alkyl radical formed during the initiation step take place efficiently.

Secondary amines for which neither carbon substituent is benzylic furnish isodiazene intermediates for which fragmentation to generate the radical pair is challenging, and unlike N-deletion with primary amines, fragmentation of the closed shell isodiazene precursor must occur for every molecule of product that is formed. At the same time, the fact that N-deletion in secondary amines takes place via the fragmentation of a closed shell precursor to a radical pair that undergoes efficient in-cage recombination enables high selectivity for the formation of intramolecular coupling products rather than a statistical mixture of intra- and intermolecular coupling products. Terminal nitrogen removal, for which an equivalent selectivity question does not arise, can profit from the reduced activation barriers for anomeric amide fragmentation through the abstraction of hydrogen atoms by escaped radicals. N-Deletion reactions of amines thus furnish an illustrative example of how a high efficiency of either cage retention or cage escape can ensure that transformations proceed with higher selectivity (secondary amines) or increased substrate scope (primary amines) than they would if cage retention was less efficient (secondary amines) or more efficient (primary amines).

### 3.2 Decarboxylative Arene Functionalization

**Figure 12:**
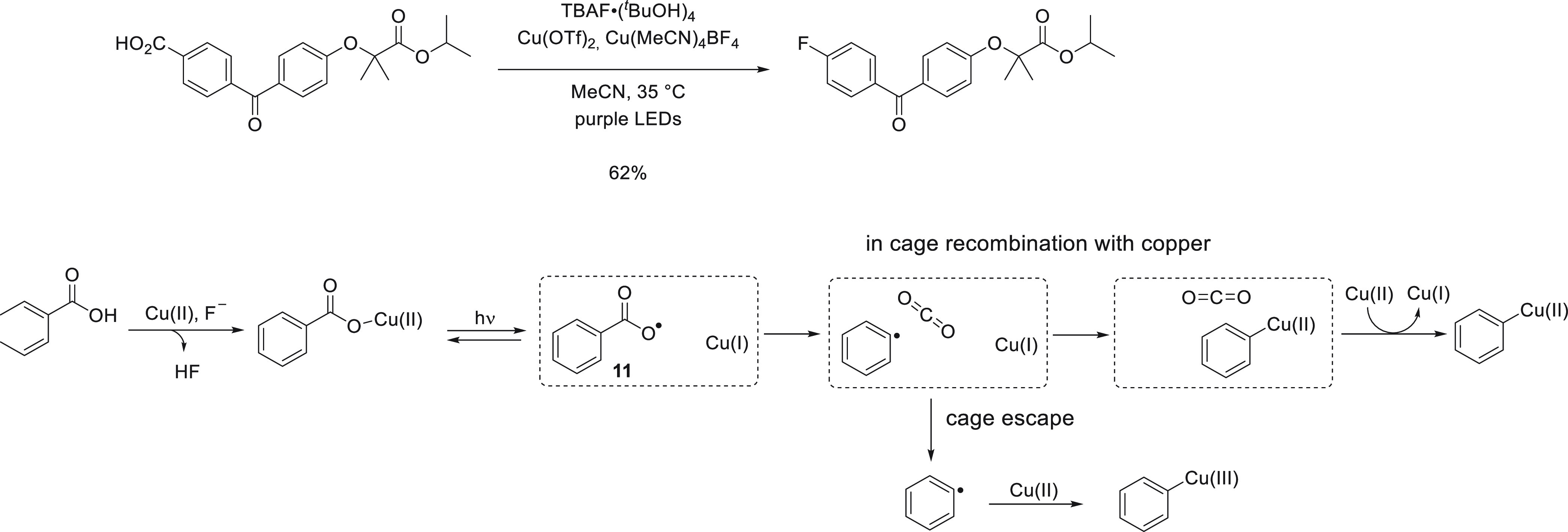
Ritter and co-workers make use of efficient in cage recombination to protect aryl carboxylate radicals from undesired side reactions.^97^

While aliphatic carboxylic acids commonly serve as precursors of alkyl radical intermediates in transformations that forge C–X bonds, aromatic carboxylic acids are more rarely employed. A substantial challenge in the case of aromatic systems is that the rate of CO_2_ release from aromatic carboxyl radicals is 3 orders of magnitude slower than those for CO_2_ loss from aliphatic carboxyl radicals (*k*_Aryl_ ≈ 10^6^ s^–1^ versus *k*_Alkyl_ ≈ 10^9^ s^–1^).^98−100^ Even though oxidative radical decarboxylation only has an activation barrier of 8–9 kcal/mol,^98^ the aryl carboxyl radical has other fast reaction pathways, such as hydrogen atom abstraction from the solvent or back electron transfer to the photocatalyst at its disposal, which can occur with rates >10^6^ s^–1^.^101,102^ In 2021, Ritter and co-workers showed that decarboxylative fluorination of aromatic carboxylic acids could be achieved with TBAF·(^*t*^BuOH)_4_ and superstoichiometric copper in the presence of light (Figure 12).^97^ Copper fulfills a 2-fold role in the reaction: (i) light-promoted ligand to metal charge transfer in Cu(II)-carboxylates permits aryl carboxyl radicals to form under mild conditions and (ii) the high-valent Cu(III)ArF complex formed after the aryl radical is recaptured by copper promotes the challenging C–F bond reductive elimination.

The presence of a solvent cage around the initially formed carboxylate radical and copper(I) favors in-cage recombination, which is an unproductive reaction (Figure 12). However, reformation of the Cu(II)-carboxylate complex permits renewed photocleavage to regenerate the carboxylate radical, while cage escape of the aryl carboxy radical would render it liable to undergo HAT in yield to ArCOOH. The need to regenerate Cu(II)-carboxylate ArCOOH would lead to unproductive consumption of the nucleophilic fluoride source TBAF·(^*t*^BuOH)_4_. For carboxylate radicals that remain confined within the solvent cage, however, the rate of decarboxylation (*k* ≈ 10^6^ s^–1^) is sufficiently fast to ensure that the generation of aryl radicals is inhibited but not fully suppressed by sharing a solvent cage with Cu(I). Furthermore, aryl radicals formed via decarboxylation reactions within the solvent cage can be rapidly captured by Cu(I) in the solvent cage and thus remain protected from unproductive side reactions. The high selectivity observed in the reported transformation shows that aryl radicals do not undergo unproductive HAT resulting in the formation of hydrodecarboxylated side products but are instead incorporated efficiently into the aryl-copper complex, which gives rise to C–F bond formation. Given the difficulty of separating arenes from fluoroarenes, even a minor contribution from aryl radicals undergoing HAT would substantially lessen the synthetic value of the reported fluorodecarboxylation, which stresses the importance of not allowing the radical intermediates to undergo cage escape. Efficient capture of carboxyl radicals by Cu(I) (which is reversible in the presence of light) favors downstream reactions of radical intermediate **11** that are possible within the solvent cage. Unimolecular CO_2_ release can effectively occur within the cage, while as long as the solvent which makes up the cage surrounding the confined radical is a poor hydrogen atom donor, HAT cannot.

### 3.3 Photochemistry of Ketones

The following section will illustrate multiple examples of how the presence or absence of a tight cage can alter and enhance the selectivity obtained in ketones photolysis reactions.^103^ The most restrictive radical cages are present in crystals, and forgoing the use of solvent has permitted transformations of complex molecules that are inefficient or unselective in solution. In the photolysis of ketones, two different reaction trajectories are commonly followed, which were illustrated in the 1930s by Norrish.^104,105^ Norrish type I reactions involve homolytic cleavage of the α-carbon bond to generate a radical pair, while Norrish type II reactions involve intramolecular abstraction of the γ-hydrogen by the oxygen atom of the photoexcited ketone to generate a 1,4-biradical. The 1,4-biradical can then either undergo β-scission to yield an olefin and a ketone, or alternatively, it can cyclize to generate a cyclobutane (Norrish-Yang reaction).^106^

**Figure 13:**
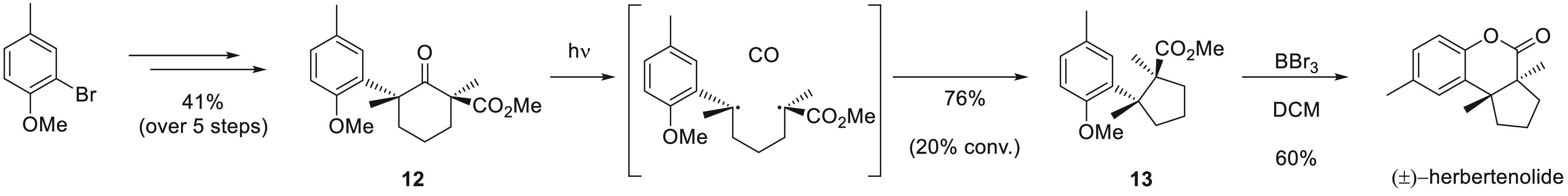
Garcia-Garibay and co-workers utilized photochemical CO extrusion in the solid state to achieve the key step in the synthesis of herbertenolide.^107^

In the synthesis of the sesquiterpene (±)-herbertenolide, the C–C bond connecting two adjacent quaternary stereogenic centers was formed in a photodecarbonylation reaction (Figure 13).^107^ Crucially, the reaction was carried out in the solid state to ensure that the stereochemical information encoded into the starting material could be efficiently transferred to the product (Figure 13). If photochemical irradiation was carried out in a 0.1 M argon-sparged benzene solution instead of in the solid state, then **12** was transformed into a complex mixture. ^1^H NMR analysis of the unpurified product indicated that none of the desired product **13** was obtained and the presence of several vinylic hydrogen signals suggested that disproportionation of the biradical intermediate constituted the dominant reaction pathway in solution. If **12** was subjected to irradiation with a medium-pressure mercury lamp in the form of a fine powder, **13** was obtained in 76% yield exclusively as the desired *trans* diastereomer. Unfortunately, the conversion of the photolysis had to be restricted to 20–35% because the energy input of photolysis led to progressive melting of **12**, which, in turn, led to side product formation. The authors highlight that their synthesis represents the first example of a solid state reaction being used as the key step in the total synthesis of a natural product.^107^ Photomediated CO extrusion takes place via electronic excitation followed by intersystem crossing to furnish a triplet that undergoes sequential α cleavage reactions within the lifetime of the triplet state. The triplet biradical resulting from CO extrusion is protected by the surrounding crystal lattice from undergoing bond rotations. Consequently, intersystem crossing to furnish a singlet biradical is followed by C–C bond formation between the two radical centers without any loss of stereochemical information.^108^

**Figure 14:**
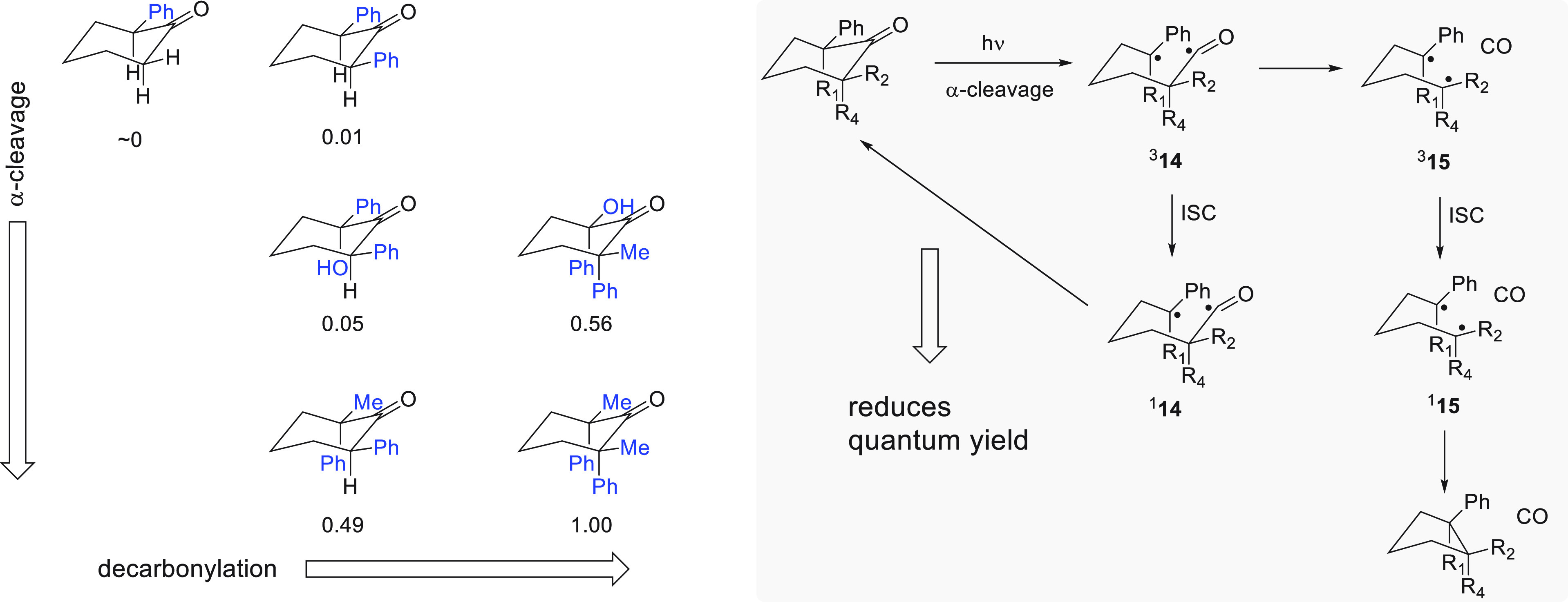
Dependence of the quantum yield (listed below structures on the left side of the figure) of CO photoextrusion from ketones in the solid state on the starting material’s substitution pattern.^109,110^

Prior work in the Garcia-Garibay group had illustrated that photodecarbonylation of crystalline ketones proceeds efficiently if radical stabilizing substituents are present on the two α positions of the ketone from which CO is extruded.^109−111^ The constrained environment of the crystal lattice ensures extremely limited mobility of the radical pairs and enables radical–radical recombination to occur chemoselectively and stereostecifically.^108^ However, the CO molecule extruded during photolysis is restricted from diffusing away from the carbon-centered radicals so that C–C bond formation between the radicals needs to kinetically outcompete reformation of the ketone. Furthermore, both α cleavage reactions that are required to release CO must occur within the lifetime of the triplet excited step: If intersystem crossing of ^3^**14** to ^1^**14** is faster than the conversion of ^3^**14** to ^3^**15**, the quantum yield is low because ^1^**14** undergoes C–C bond formation to reform the starting material with high efficiency. The yields for photochemical CO extrusion in the solid state thus depend strongly on the α substitution pattern of the ketone, because the nature of the substituents affects the rate of α-bond cleavage for triplet ^3^**15** (Figure 14).^109,110^ In solution, on the other hand, all compounds shown in Figure 14 undergo reactions with quantum yields >0.6 to yield mixtures of recombination and disproportionation derived products.

**Figure 15:**
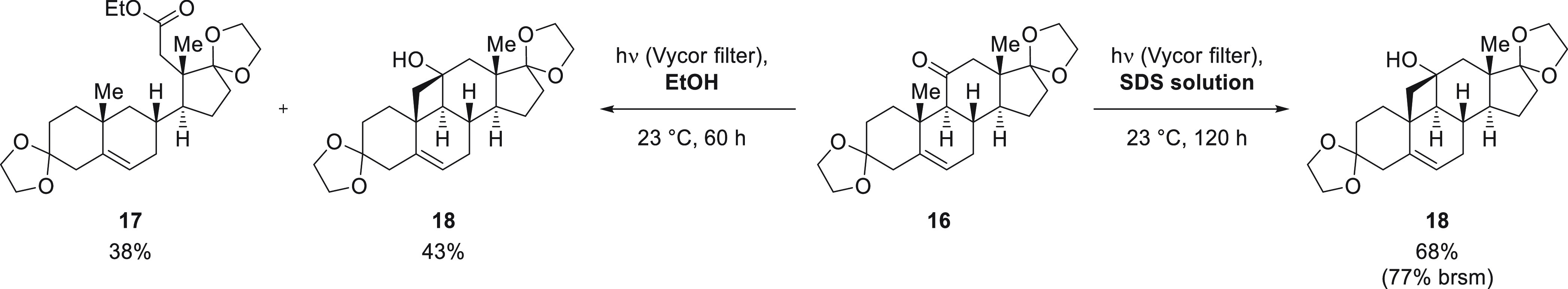
Baran and co-workers favored Norrish type II over type I cleavage in the synthesis of ouabagenin by carrying out the photolysis of **16** in the solid state.^112^

In their concise synthesis of the polyhydroxylated steroid ouabagenin via an approach reliant on redox relay and oxidative stereochemistry relay, Baran and co-workers made use of a Norrish type II reaction in the solid state (Figure 15).^112^ Ketalization of commercially available cortisone acetate followed by recrystallization furnished **16**, which was irradiated with a mercury lamp in the presence of SDS and water to furnish **18** in 68% yield, along with 12% recovered starting material **16**. While the photochemical transformation in the solid state is slow, likely due to limited surface area of the microcrystalline solid, it furnishes substantially higher selectivity for the formation of **18** compared to the corresponding reaction in benzene solution. In solution, Norrish type I cleavage of the C9–C11 bond in the steroid framework gave rise to 38% ester **17**, while a Norrish–Yang reaction was responsible for the formation of desired alcohol **18** in 43% yield.^112,113^

**Figure 16:**
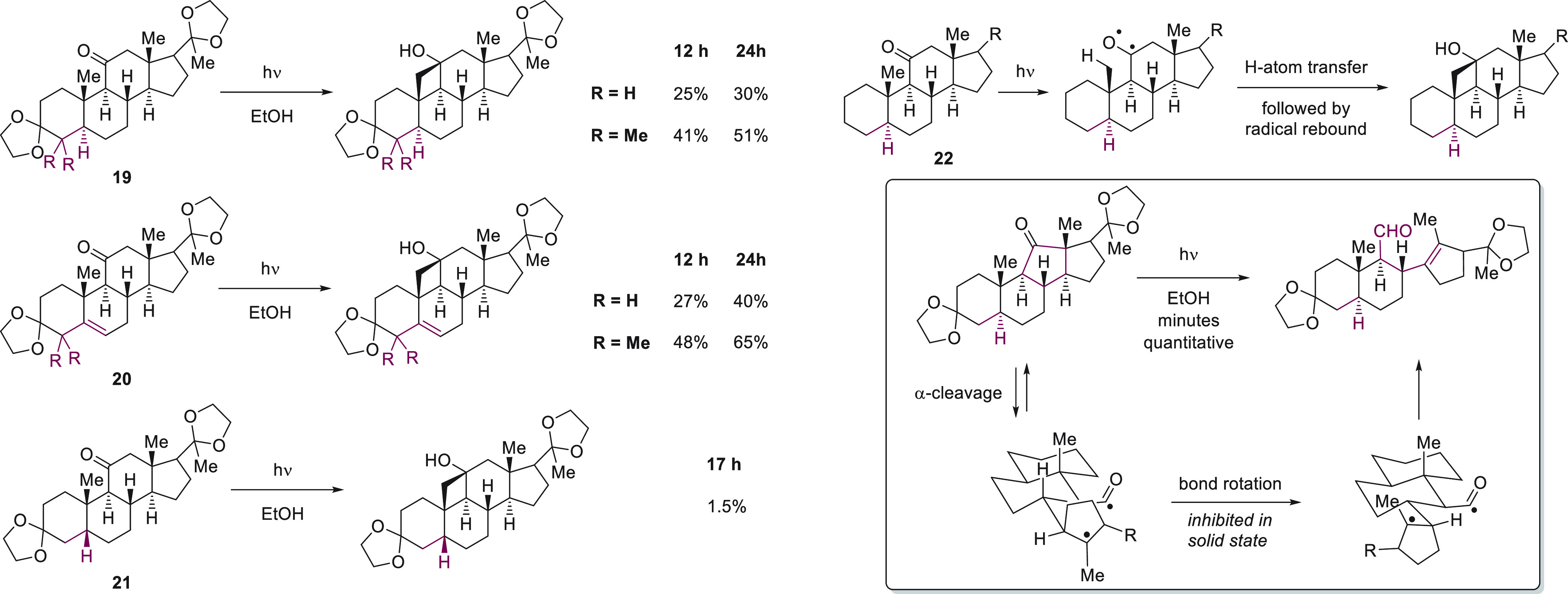
Detailed investigations of the behavior of steroidal ketones during photolysis laid the foundation for later applications to total synthesis.^114−121^

Jeger and co-workers carried out in depth studies of the photochemistry of ketones with similar structures to **16** in ethanol solution, which inspired the disconnection used by Baran and co-workers.^113−121^ The Jeger group found that the presence or absence of unsaturation as well as the configuration at positions distant from the reaction site (marked in red in Figure 16) had a notable influence on the efficiency with which cyclobutanol products were formed. The Norrish type II photoreaction proceeds via the formation of an activated carbonyl intermediate, which can be represented as a diradical, followed by H atom transfer and radical–radical recombination.^122^ Substrates that undergo cyclobutanol formation efficiently are those for which the methyl group that undergoes H atom abstraction is situated close to the carbonyl oxygen in the starting material. The incorporation of distal dimethyl substitution for **19** as well as **20** leads to notably higher yields because the reactive centers are pushed closer to one another in the starting material.^119^ Switching from the trans ring junction in **19** to the cis ring junction in **21**, on the other hand leads to a conformationally less rigid starting material for which the reactive centers are further separated in the starting material, so the yield for Norrish type II photoreaction is reduced to 1.5%.^117^ The Jeger group also studied photorearrangement for **22**, in which one of the cyclohexyl rings is replaced by a cyclopentyl unit.^120^ The change in ring size not only increases the distance between the carbonyl oxygen and the methyl group β to the carbonyl but also increases the amount of strain present in the carbonyl-containing ring. Consequently, photoexcitation of the carbonyl is followed by rapid α-fragmentation to generate a pair of carbon-centered radicals (Norrish type I process). Rotation around a C–C bond positions the diradical intermediate for a facile disproportionation reaction that furnishes an aldehyde and an olefin.

Work from the Jeger group thus highlights structural aspects of steroidal ketones that favor Norrish type I versus type II behavior. While all their experiments were carried out in solution, the C–C bond rotation required to access product **22** illustrates how the presence of a tighter solvent cage can eliminate contributions from Norrish type I reactions and thus access increased yields for substrates for which both processes are inherently viable reaction pathways: α-cleavage following carbonyl group excitation is a reversible process, so if bond rotation does not occur, the diradical intermediate will revert to the starting ketone.

**Figure 17:**
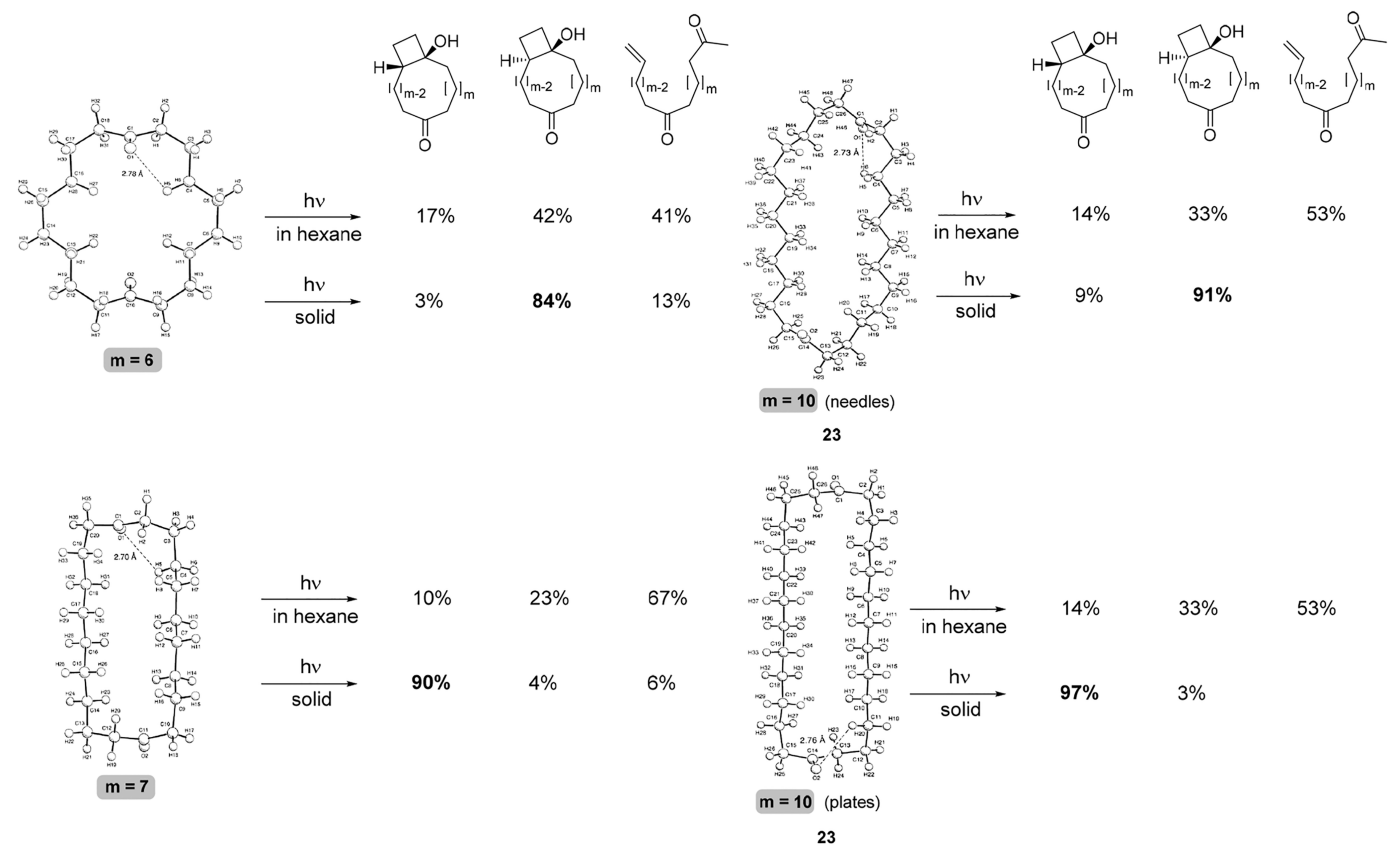
Drastic changes in the selectivity for the formation of different photolysis products of diketones were observed by Scheffer and co-workers in solution and in the solid state.^123^ Adapted duced from ref (123). Copyright 1996 American Chemical Society.

In 1995, the Scheffer group applied the crystal structure–reactivity correlation method to the photochemistry of a series of medium-sized ring and macrocyclic diketones.^123^ Analysis of the distance between the carbonyl oxygen atom and the neighboring γ-hydrogen atoms was used to predict the likelihood of Norrish type II photoreactions in the solid state, which were then compared with the observed reaction outcomes in the solid state and in solution. Striking differences in the selectivity could be achieved depending on whether the reaction was carried out in solution or in the solid state (Figure 17). Notably, diketone **23** could be crystallized as two different polymorphs (needles or plates), and cyclobutanols with either a *cis* or a *trans* ring junction could be accessed selectively depending on which of the two γ-hydrogen atoms was positioned closer to the carbonyl oxygen in the crystal structure (Figure 17).

Ketone photolysis of both the needle and the plate-shaped polymorph of **23** showed a decrease in selectivity as the reaction temperature was gradually elevated. For the needle shaped polymorph, the dominant product isomer was formed in 93% (0 °C), 91% (20 °C), 89% (40 °C), and 83% selectivity (65 °C), but the selectivity never declined to the values obtained in solution. The authors concluded that the reaction cavity provided by the crystal maintained its anisotropic shape even though 65 °C is very close to the melting point of diketone **23** (70 °C).^123^ The substantial effect of proximity and dynamics on the reactivity of radical pairs is borne out by the fact that the dominant product obtained in the solid state is derived from abstraction of the H atom marked in Figure 17 for each of the structures. The H atom in question is located close to the ideal distance of 2.72 Å away from the carbonyl oxygen, which corresponds to the sum of the van der Waals radii of the abstracting and abstracted atoms. The analogous photochemical reaction in hexane solution proceeds via a diradical intermediate that is not confined to a particular conformer via a rigid cage, and the increased mobility of the radical intermediate gives rise to a mixture of products (Figure 17).

### 3.4 Fragment Coupling

**Figure 18:**
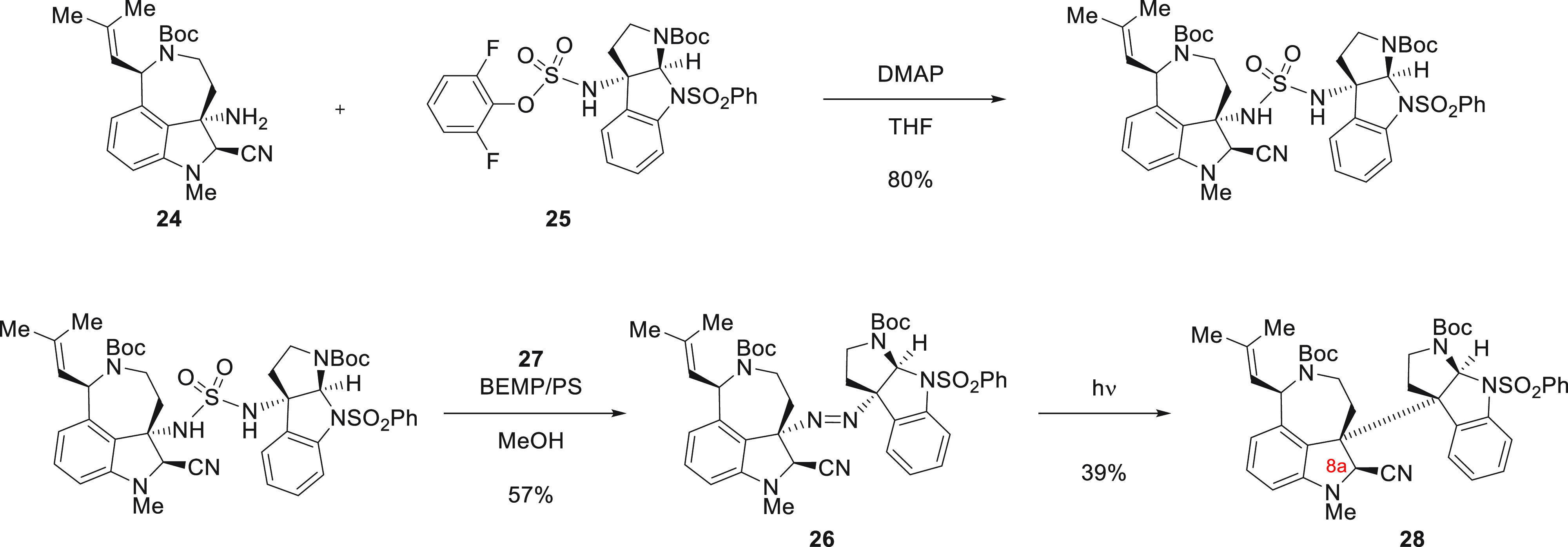
Fragment coupling via radical–radical combination in the solid state.^89^

The total synthesis of (−)-communesin F by Movassaghi and co-workers features an heterodimerization step that brings together two complex fragments at a late stage of the enantioselective total synthesis (Figure 18).^89^ Importantly, the fragment coupling step needed to occur in a manner that established the correct stereochemistry at the two adjacent quaternary centers. In an attempt to utilize a diazene strategy for the generation of radical pairs the Movassaghi group had previously employed to achieve the coupling of complex fragments, tricyclic amines **24** and **25** were prepared.^124−126^ The two fragments **24** and **25** could be brought together in the form of a sulfamide in 80% yield on a gram scale. Extensive optimization was required to achieve the subsequent conversion of the sulfamide to diazene **26** in a chemoselective manner, since competing arene halogenation was observed for a large number of the reaction conditions that were attempted. A combination of *N*-chloro-*N*-methylbenzamide (**27**) and polystyrene-bound 2-*tert*-butylimino-2-diethylamino-1,3-dimethylperhydro-1,3,2-diazaphosphorine (BEMP) furnished **28** in 57% yield. Photochemical nitrogen expulsion from the diazene was carried out for a thin film of **26** coating the bottom of a round-bottom flask, which furnished radical pairs that recombined to yield heterodimer **28** in 39% yield. The high level of diastereoselectivity of the radical coupling was ascribed to both the speed of radical–radical recombination in the solid state reaction and the directing influence of the C8a-nitrile.

### 3.5 Deoxygenative Alkylborylation of Aldehydes

**Figure 19:**
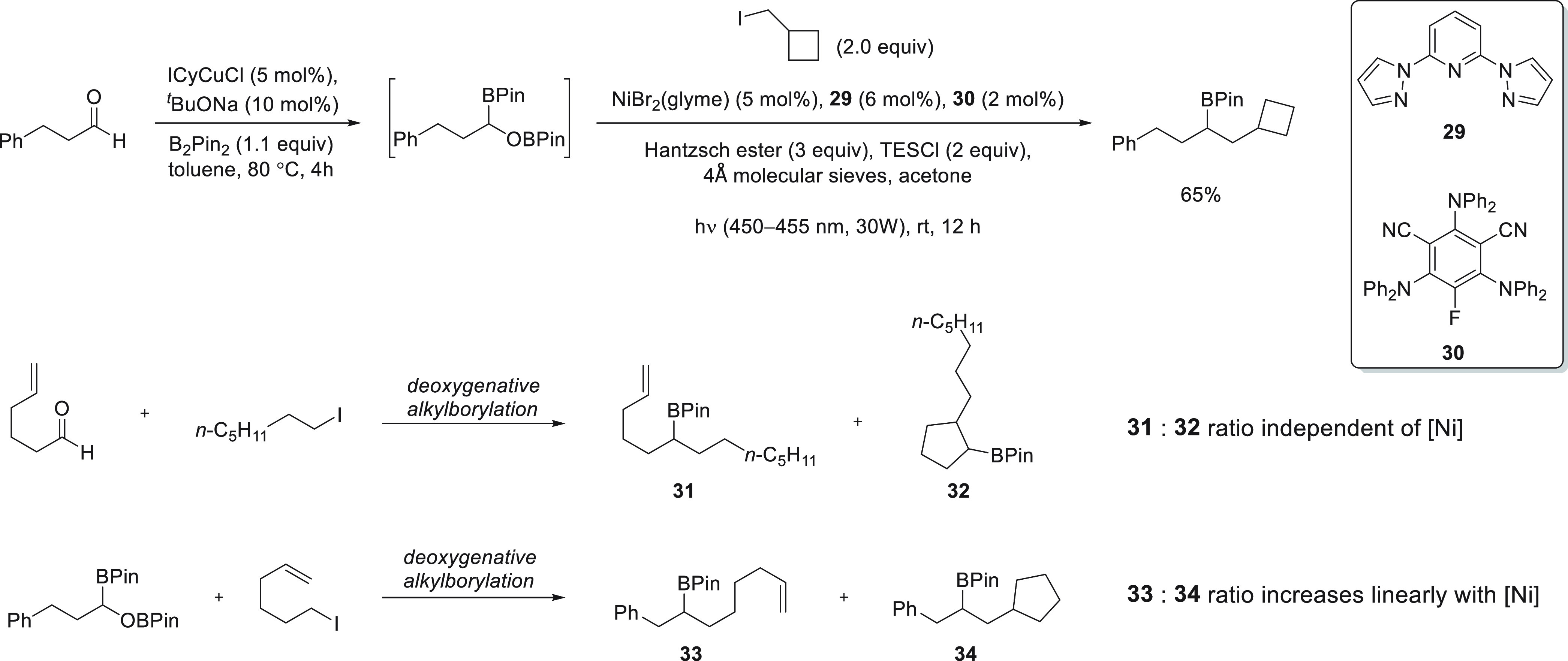
Two radical intermediates with different lifetimes participate in the deoxygenative alkylborylation of aldehydes.^127^

In 2022, Xu and co-workers reported a deoxygenative alkylborylation of aldehydes to access α,α-dialkylboronates with a dual catalyst system comprising NiBr_2_(glyme), ligand **29**, and organic photoredox catalyst **30**.^127^ Mechanistic work showed that both the alkyl iodide and the aldehyde reaction partners give rise to radical intermediates over the course of the reaction and that the lifetimes of the two radicals are substantially different. Prior to the nickel catalyzed reaction, the aldehyde reaction partner is subjected to copper catalyzed diborylation. Oxidative addition of the Ni(I) catalyst into the C–OBPin bond furnishes a nickel alkyl intermediate that is able to undergo reversible Ni–C bond scission to furnish a caged radical pair. The alkyl iodide reaction partner, on the other hand, reacts with the excited photocatalyst to generate a cage-escaped primary alkyl radical that is subsequently captured by the nickel catalyst. The authors demonstrate the different radical lifetimes via a straightforward and ingenious mechanistic experiment, wherein the ratio of cyclized and linear products of aldehyde- and iodide-containing radical clock substrates was monitored as the Ni catalyst loading was varied (Figure 19). Since an increase in the nickel concentration leads to a reduced lifetime of the free radical derived from the alkyl iodide coupling partner before it is captured by nickel, an increased amount of noncyclized product **33** is detected when then concentration of nickel rises. In the case of the aldehyde-derived radical clock substrate, no change in the ratio of **31** to **32** results from a variation in the nickel catalyst loading because radical formation and recapture occurs within a solvent cage.

### 3.6 Direct Partial Oxidation of Methane

The walls of a pore in a zeolite or the surface of a support material provide a level of confinement to radicals that is less stringent than that experienced by radicals inside nonporous crystalline materials, but more severe than that offered by solvent cages. Surfaces and pores thus allow radicals to sample a diffusional regime similar to that offered by enzyme pockets, a valuable tool both for understanding as well as translating cage effects operative in enzymatic reactions to synthetic systems. Inspired by nonheme iron-oxo enzymes, Solomon and Schoonheydt reported an iron-modified zeolite catalyst for which C–H hydroxylation proceeded via HAT from methane to the Fe(IV)=O center followed by rapid radical rebound.^128^

**Figure 20:**
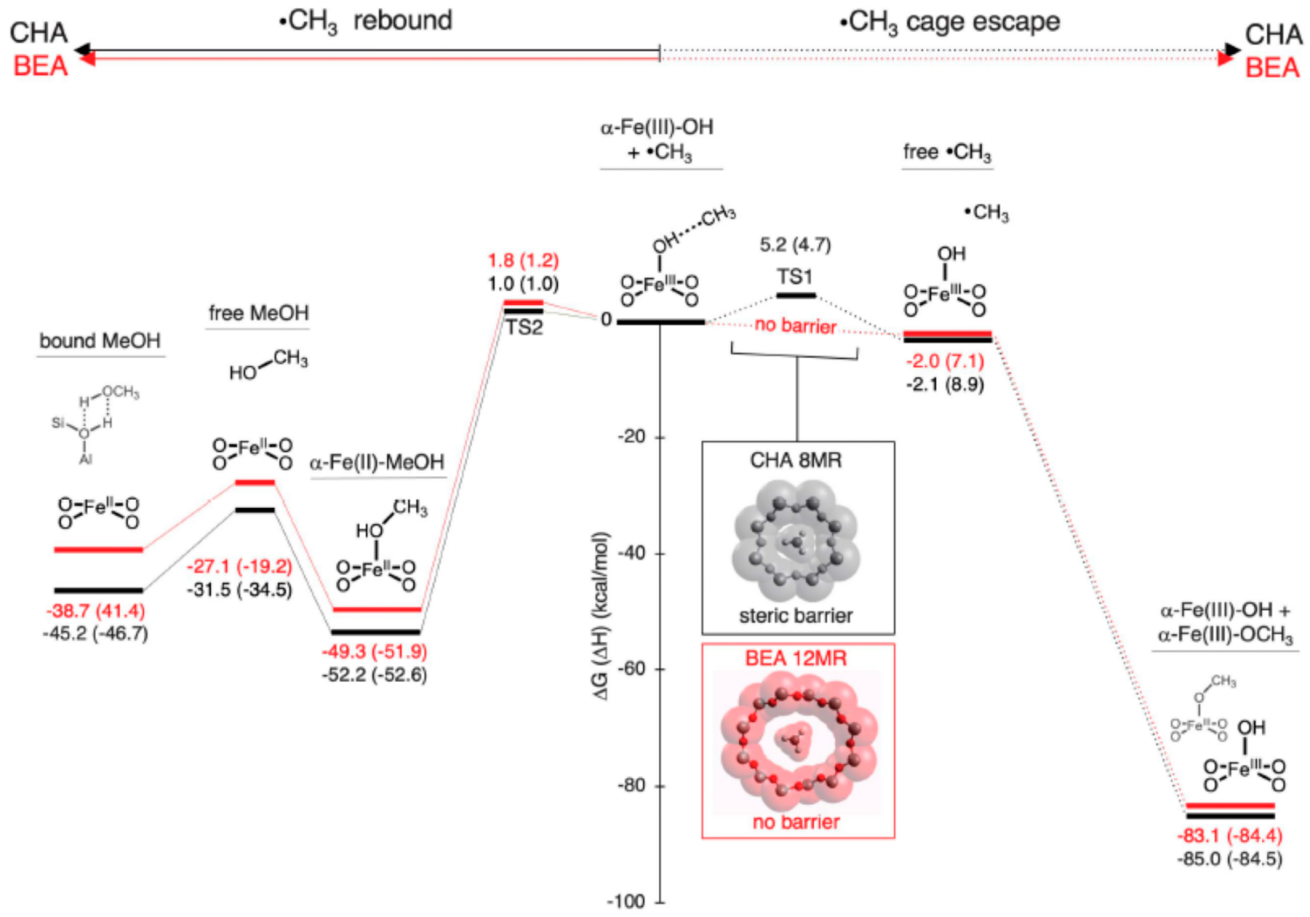
Small zeolite pore apertures restrict the ability of methyl radicals to diffuse into adjacent zeolite cages and react with Fe(IV)=O, a pathway that results in the inability of the methane oxidation catalyst to turnover.^128^ Reproduced with permission from ref (128). Copyright 2021 AAAS.

Escape of the radical from the vicinity of the iron site after HAT and migration to another iron-containing zeolite cage can give rise to a barrierless reaction between the methyl radical and Fe(IV)=O to form Fe(III)–OMe, while Fe(III)–OH is left behind in the cage in which HAT took place (Figure 20). Neither Fe(III)–OMe nor Fe(III)–OH can be efficiently regenerated to Fe(II), the precursor for Fe(IV)=O, so that each methyl radical cage escape leads to the deactivation of two catalytic sites. Solomon and Schoonheydt compared the performance of Fe(IV)=O sites installed in either zeolite BEA or zeolite CHA. The inner diameter of the iron containing cages is the same for both zeolites, but the diameters of molecules that can freely diffuse through the windows that link the individual cages is reduced from 5.9 Å for BEA to 3.7 Å for CHA. The reduction in window size in going from BEA to CHA introduces a diffusion barrier of 5.2 kcal·mol^–1^ for cage transit of the methyl radical (Figure 20). The kinetic diameter of methane has been determined to be 4.1–4.2 Å, which is in line with the observation that transit of the methyl radical between different cages is rendered challenging in CHA. Solomon and Schoonheydt showed by detailed Mössbauer studies that the increased cage efficiency for the methyl radical rebound reaction for CHA led to the reformation of 37% Fe(II) sites after one catalytic cycle and a total two-cycle methanol yield that corresponded to 140% of the one-cycle yield. Use of zeolite BEA, on the other hand, ensured that nearly all catalytic centers are deactivated by a single catalytic cycle and require reactivation by steam treatments and high temperature reduction in order to recover their ability to hydroxylate methane.

### 3.7 Late-Stage Methylation

Cage effects not only are significant when pairs of radicals are considered but also affect propagation steps in which a radical reacts with a closed shell species. The cage model postulates a longer contact time between reactants than that predicted by the free-diffusion model.^130^ The selectivity with which reactive radicals can discriminate between different reaction partners is much reduced in aqueous solution, for example, while the selectivity of HAT at different positions within the same molecule is relatively unaltered. A cage model can account for differential selectivity control as the medium is varied because prolonged encounters lead to efficient reaction of the radical even with the less reactive species in solution, while the solvent cage is commonly sufficiently flexible to allow the reaction partner to rotate to expose its most reactive C–H bond.^130^ The solvent cage in propagation reactions is assembled after the initial radical formation step, and precise studies of how it influences reactivity are thus more complex.

**Figure 21:**
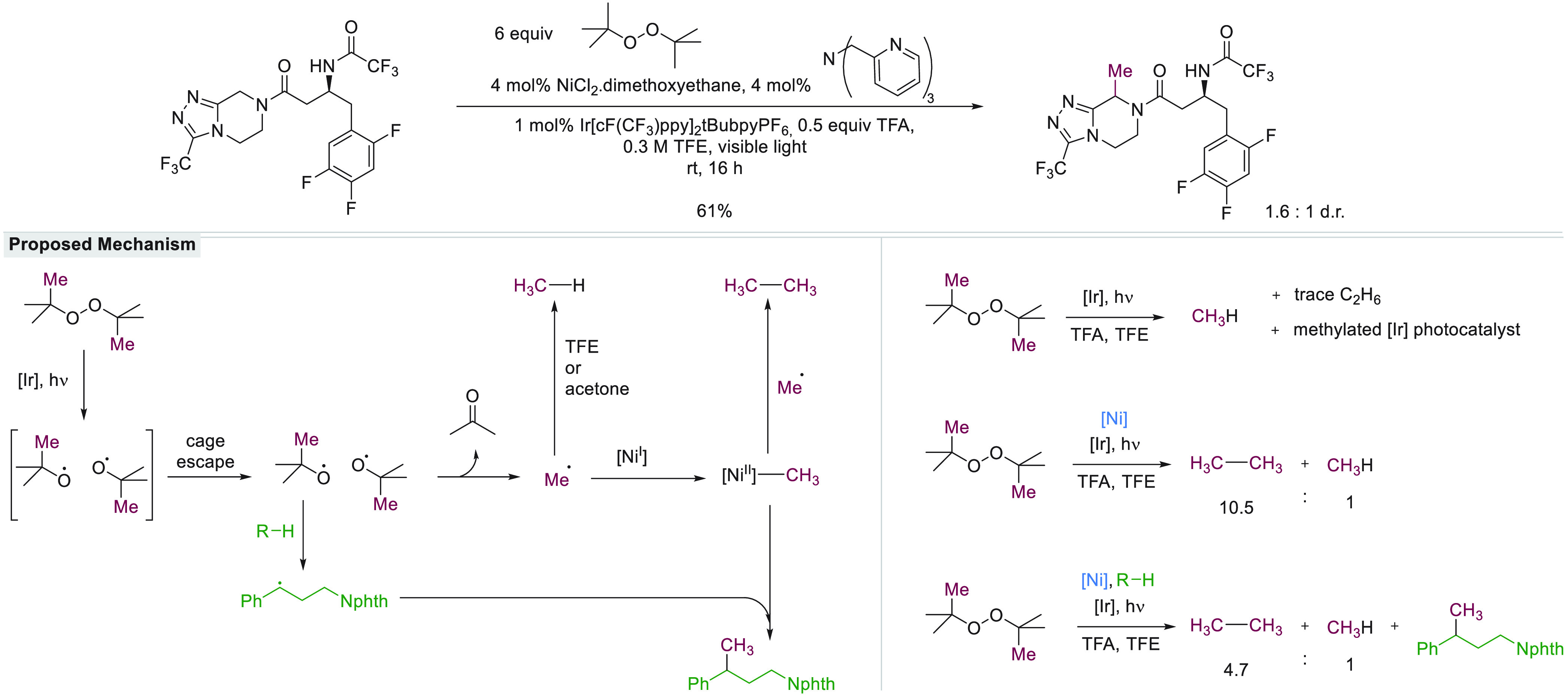
Stahl and co-workers developed a nickel-catalyzed and light-mediated transformation for the introduction of methyl groups into complex substrates.^129^

Since radicals are commonly generated via homolytic bond cleavage, they are generally produced in pairs. To undergo reactions with a specific reaction partner in solution, a radical needs to be able to escape radical recombination inside the solvent cage in which it was generated and survive sufficiently long in solution to encounter the designated reaction partner. Ensuring a selective intermolecular reaction between a reagent that is not present in solvent quantities and a radical is particularly challenging for highly reactive radicals, such as simple methyl radicals. In 2021, Stahl and co-workers were able to demonstrate late-stage methylation with methyl radicals by capture of both the methyl radical and a substrate-derived radical using a nickel catalyst, which subsequently enables C–C bond formation via reductive elimination (Figure 21).^129^ The key reagent in their nickel- and light-mediated reactions is di-*tert*-butyl peroxide, which undergoes light-mediated homolysis to furnish a pair of alkoxylradicals. Alkoxylradicals are known to undergo two reaction pathways, hydrogen atom abstraction and β-methyl scission, the relative importance of which depends on the reaction conditions. Interestingly, the nickel-catalyzed methylation reaction relies on the occurrence of both pathways with carefully balanced relative rates: hydrogen atom abstraction by the alkoxyl radical generates a radical intermediate from the reaction substrates, while β-methyl scission yields the methyl radical which is incorporated into the reaction product. Formally, the desired product is formed via the combination of two radicals, but direct reaction between the two radical fragments would not be kinetically viable due to the low concentration of both radicals. To circumvent this problem, Stahl and co-workers included a nickel catalyst, which efficiently captures the methyl radical and generates a Ni(II)-Me intermediate with a sufficiently long lifetime that encounters with the substrate derived radical becomes feasible (Figure 21). A number of complex molecules are thus able to undergo C–H methylation with methyl radicals derived from a relatively inexpensive reagent. Late-stage methylation is an example of a transformation where efficient cage escape is desirable because efficient hydrogen atom abstraction from the substrate requires that a sufficient fraction of the alkoxyl radical is able to escape its solvent cage prior to undergoing β-methyl scission to yield a methyl radical.

### 3.8 HAT with Metal Hydrides

Hydrogen atom transfer reactions involving metal hydrides (MHAT) can take place via different mechanisms depending on whether the metal forming the metal hydride is supported by strong or weak ligand field ligands. For metal hydrides based on Mn, Fe, or Co surrounded by weak-field ligands, the metal hydride complexes show low stability and undergo swift hydrogen atom transfer even with unactivated substrates such as alkenes.^5,131,132^ Typically silane or borohydride reductants are employed which provide the thermodynamic driving force for the formation of the highly reactive metal hydride from M–O or M–F.^133^ Weak field MHAT catalysts commonly give rise to reactions in which the radicals that are generated undergo efficient cage escape: hydrogen atom transfer from the metal-hydride to the substrate is thermodynamically highly favorable, so that radical–radical recombination to reform M–H and the olefin is unusually inefficient. Despite the fact that reactions proceed via cage escaped free radicals, weak field MHAT catalysis can furnish the desired hydrofunctionalization products in high selectivity, as long as the free alkyl radicals do not react efficiently with either the solvent or the terminal oxidant. Crucially, the concentration of M–H in solution is low (slow formation due to the weak M–H bond), as is the concentration of the alkyl radical, which disfavors recombination reactions between alkyl radicals. Consequently, the alkyl radical reacts with high selectivity with the stoichiometrically added trapping reagent.

For MHAT catalysts in which the metal center is surrounded by strong field ligands, hydrogen-atom transfer from M–H to a substrate tends to be endothermic, as is commonly the case for reaction steps in which radical pairs are generated from closed shell species. Therefore, reverse hydrogen atom transfer from the radical pair of the metal readily takes place and notable cage effects can be observed. CIDNP effects were observed for the strong field MHAT catalysts such as Mn(CO)_5_H, Co(CO)_4_H, and Fe(CO)_4_(SiCl_3_)(H), which confirmed that hydrogen atom transfer to olefin substrates was a reversible step that leads to the generation of radical pairs.^134−137^ Experiments with solvents of different viscosity demonstrated that cage efficiencies for MHAT reactions tend to be higher than those observed with most organic radicals,^138,139^ which is likely due to the high inertia of the large organometallic fragments and efficient ISC for metalloradicals.^5,58^ The low fraction of radicals that escapes the solvent cage in MHAT is a special case of the general observation that homolytic cleavage of metal–ligand bonds in solution generally is substantially influenced by persistent solvent cages (see section 4.1). A more thorough discussion of mechanistic insights into MHAT reactivity, including the crucial role of caged radical pairs, can be found in an excellent perspective article on the topic by Shenvi, Holland, and co-workers.^5^

### 3.9 Other Recent Examples of Cage Effects in Synthetic Chemistry

In 2022, Lindsay and co-workers reported a formal [3,3]-sigmatropic rearrangement of Breslow intermediates that proceeds via a close radical pair and thus generates products in a regio- and diastereoselective manner.^140^ The transformation provides access to hindered homoallylic alcohols and can be used to forge one of the C–C bonds that extends from an all-carbon quaternary center. While the addition of TEMPO has only a minor effect on the efficiency of product generation, more reactive radical traps such as the 2-methyl-2-nitrosopropane dimer substantially reduced the yield of the rearrangement product, which points to the intermediacy of a short-lived radical pair. Diastereomeric ratios ranging from 1.2:1 to 5:1 further support a radical mechanism, since concerted [3,3]-sigmatropic rearrangements are known to be stereospecific.

Chen, Koh, and co-workers demonstrated that stereoselective synthesis of C-glucosides via the intermediacy of glycosyl radicals can be promoted by a simple iron salt in the presence of a stoichiometric reductant.^141^ While a number of substrates could be accessed via simple capture of the cage-escaped radical by a suitable electrophile, a substantial extension of the substrate scope was possible due to the development of a secondary set of reaction conditions that include a nickel catalyst in addition to the iron catalyst. C-glycoside synthesis thus constitutes another example of the suitability of nickel catalysts for the capture of cage-escaped radicals and their conversion into synthetically valuable products via nickel-mediated cross coupling chemistry.

Primary amines are commonly present in natural products and pharmaceuticals, but unstrained C–N bonds are rarely used as synthetic handles for the formation of C–C bonds via cross-coupling reactions. Michaudel and co-workers demonstrated in 2023 that amines could be converted into diazenes via sulfur(VI) fluoride exchange (SuFEx) click chemistry followed by oxidative extrusion of SO_2_.^142^ Fragmentation of the diazene intermediate via energy transfer from an excited iridium photocatalyst led to alkyl radical intermediates that were captured by a nickel catalyst, which promoted C(sp^3^)–C(sp^2^) bond formation. Unlike radical–radical fragment coupling from diazene precursors employed by Movassaghi and co-workers in total synthesis endeavors (section 3.4), which relies on efficient in-cage recombination, Michaudel and co-workers capture cage-escaped radicals derived from diazenes in order to carry out cross coupling reactions.

In 2022, Nagib, RajanBabu, and co-workers showcased γ-selective desaturation and C–H functionalization of amines via a triple H atom transfer cascade.^143^ In order to make use of metal H atom transfer as a mild and robust way of accessing C-centered radicals, the amine substrates were outfitted with an olefin-containing protecting group. Following the generation of a C-centered radical situated on the amine protecting group, 1,6-hydrogen atom transfer places the radical center on the substrate carbon atom that is situated in the γ-position of the amine. H atom transfer to the cobalt MHAT catalyst leads to the generation of the γ-unsaturated protected amine. The authors propose that 1,6-HAT is followed by δ-MHAT rather than β-MHAT to generate contra-thermodynamic desaturation due to the faster rate of H atom transfer from the sterically less hindered δ-position. Crucially, Nagib and RajanBabu showed that the use of an excess of a suitable scavenger leads to the formation of γ-functionalized rather than γ-unsaturated amine products. Because 3 equiv of TsCl or TsCN can induce chlorination or cyanation, respectively, the amine substrate radical is able to undergo reasonably efficient cage escape. Considering the low BDE of Co–H for the high field cobalt salen complexes used as MHAT catalysts, H atom transfer from the substrate radical to Co(II) is expected to be slow, which is in line with the observed long lifetime of the amine-derived radical and its ability to escape the solvent cage.

Martin and co-workers recently disclosed a base-mediated α-difluoroalkylation of benzyl amines in which C–C bond formation occurs via radical–radical recombination.^144^ SET between trifluoromethyl-arenes or–heteroarenes and an imine derived from the amine substrate furnishes the radical pair which recombines to yield fluorocarbon substituted amines. Despite the presence of a radical pair, only the imine-derived TEMPO adduct could be isolated when 2 equiv of TEMPO was added to the reaction conditions. Additional spin-trapping experiments with PBN were carried out to confirm the presence of the radical intermediates via characterization by EPR.

## 4 Cage Effects as a Source of Error

### 4.1 BDE Determination

**Figure 22:**
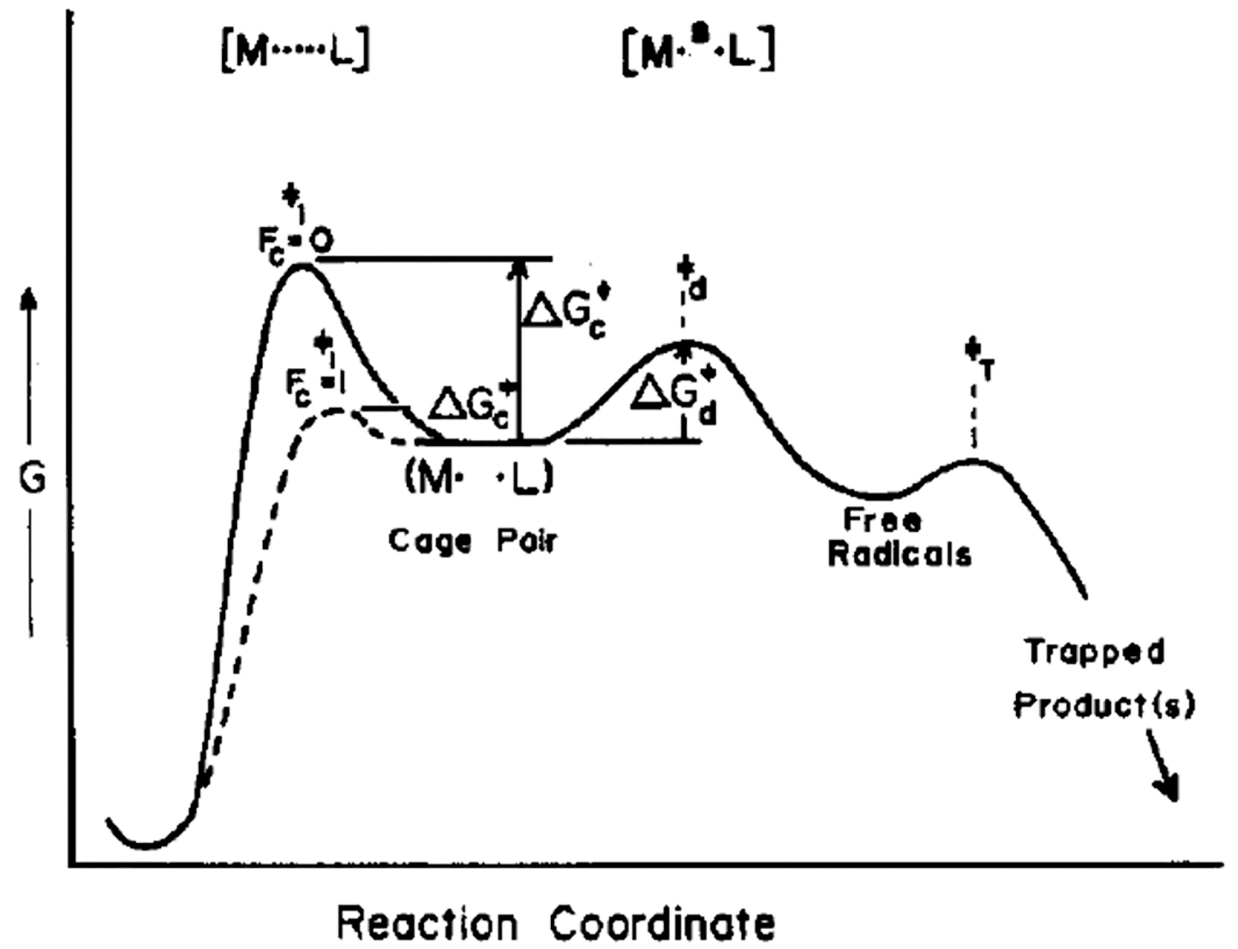
Not only the strength of the metal–ligand bond but also the cage efficiency determine BDE values determined in solution. Reproduced with permission from ref (145). Copyright 1988 Elsevier.

The bond dissociation energy (BDE) is defined as the enthalpy change that accompanies gas-phase homolytic bond dissociation at 298 K. However, the limited volatility of many compounds in the temperature range in which they are chemically stable results in the determination of many BDE values, especially for organometallic complexes, from experiments carried out in solution. In addition to accounting for any specific solvation of free radical products in solution that would ensure substantially larger stabilization of the product radicals than the starting complex, working in solution also requires one to disentangle potential contributions from cage escape to the observed reaction enthalpy for bond dissociation (Figure 22). Experimental BDE determination relies on the capture of the released radicals by a suitable trapping reagent, so the energy that needs to be supplied to generate the product is the sum of the real BDE and the activation energy for radical cage escape. Cage efficiency factors as well as the activation parameters of in-cage recombination and cage escape must therefore be known to accurately determine BDE values for homolytic bond cleavage reactions in solution.^145^ Furthermore, variation of the cage efficiency with temperature must be understood before the activation energies for bond cleavage can be extracted from the temperature dependence of the observed rates of bond homolysis.

Endicott and co-workers determined via picosecond flash photolysis of the homolysis of the Co–C bond in coenzyme B12 in water that the rate constant for recombination (*k*_c_) is 1.3 × 10^9^ s^–1^ while the rate constant for cage escape is *k*_d_ = 0.5 × 10^9^ s^–1^, from which one can calculate a cage efficiency of *F*_c_ = *k*_c_/*k*_d_ ∼ 0.7.^146^ Cage escape is an activated process so that the experimentally determined energy determined to furnish the trapped products is not due to the energy requirement of Co–C bond cleavage but rather the sum of energy required for homolytic bond cleavage and the energy required for the carbon radical to escape the solvent cage. Consequently, the observed enthalpy for homolytic bond cleavage in the presence of free radical scavengers was found to increase as the solvent viscosity increased because a higher solvent viscosity led to a higher energy requirement for cage escape.

### 4.2 Failure of Radical Clocks

The groups of Lipscomb,^147^ Groves,^65,148^ and Lippard^149,150^ found that the use of radical clock substrates to evaluate the lifetime of radical intermediates in reactions with soluble methane monooxygenase (sMMO) or P450 enzymes is challenging: the resulting product mixtures can be highly complex, the occurrence of rearranged products may fail to correlate with chemical nature of C–H bond being broken or the magnitude of the rate constant for rearrangement of the radical clock substrate. Instead, substrates that bear bulky substituents in close proximity to the C–H bond undergoing cleavage gave rise to longer-lived radicals that rearrange, while substrates with a slimmer steric profile did not yield rearranged products.^147^ Both the speed of the ring opening of the radical intermediate and its fit within the enzyme’s substrate pocket thus determine whether rearrangement is observed or not.^151^

**Figure 23:**
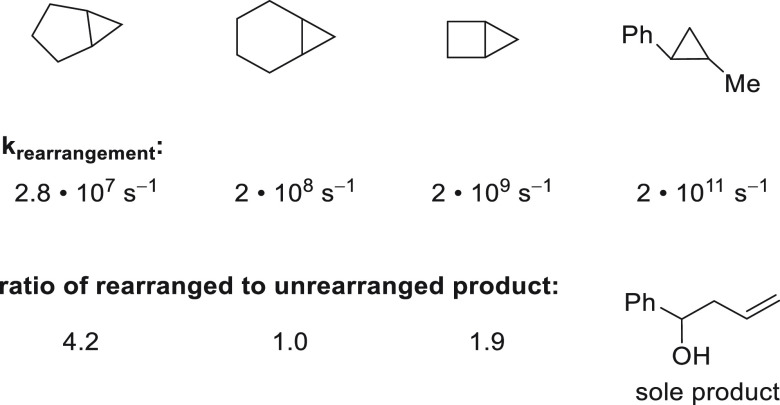
Extent of rearrangement of radical clock substrates during oxidation by diiron oxygenase enzyme suggests apparent radical lifetimes of 0.78–170 ns.^62,152,153^

A particular complication arises when the rate constant for rearrangement of a radical clock substrate is of the same order of magnitude as the rate constant for cage escape.^6,152^ Under these circumstances, essentially all escaped radicals undergo rearrangement, while all in-cage reactions give rise to nonrearranged product, and the observed outcome of the radical clock experiment measures the cage efficiency rather than the lifetime of the radical intermediate.^6^ When performing a radical clock experiment, we generally think of the radical intermediate as having a single average lifetime, but if not all radicals either remain inside the cage or escape the cage (0 < *F*_c_ < 1), two distinct populations of radical intermediates are present during the reaction: those that escape and those that do not. The lifetime of the two populations can be substantially different from one another so that radical clock substrates spanning a range of different lifetimes can give rise to partial rearrangement. Groves and co-workers investigated hydrocarbon hydroxylation with the enzyme AlkB, for which both rearranged and unrearranged products were observed for clock substrates spanning 2 orders of magnitude in their rearrangement rates (Figure 23). The ultrafast probe *trans*-1-methyl-2-phenylcyclopropane only furnished rearranged product, however, which showed that a rearrangement rate of 2 × 10^11^ s^–1^ was sufficiently fast to kinetically outcompete both cage escape and in-cage recombination.^62^ Both cage escape and in-cage recombination are estimated to occur with rate constants of ∼10^10^ s^–1^ so that for the three slower probes in Figure 22 a portion of the radicals recombines inside the cage to yield nonrearranged products, while a second portion escapes the cage. The rate constant for the hydroxylation of solvent separated radicals is slow compared to the rearrangement of even the slowest probe, so that all three probes yield a fraction of rearranged product.^152^

### 4.3 Misassignment of Reaction Mechanisms Based on Stereochemical Outcome

If a C–X bond is transformed in a highly stereoselective fashion into a C–C bond, the intermediacy of a carbon radical is frequently discounted if the carbon radical is able to access a low-energy conformation that is close to planar. However, if the interconversion of C–X to C–C takes place via caged radicals, the radical intermediate may be sufficiently short-lived to ensure that the stereochemical information present in C–X is passed on to the product (see section 2.4). 1,2-Wittig rearrangements are known to proceed via radical pairs, but they can nonetheless show high levels of stereoselectivity, so that a mechanistic assignment based on stereochemical outcome alone is open to misinterpretation.^154,155^

## 5 Outlook

Cage effects are a constant and often underappreciated factor that affects the outcome of all radical reactions that are carried out in condensed phases. Irrespective of whether a high or low cage efficiency is desired for a given transformation, an appropriate reaction medium can lead to substantial yield or selectivity gains by controlling the dynamics of radical intermediates during the first nanosecond after their generation. The influence of the medium on selectivity can be readily appreciated by comparing the results of photo-Fries rearrangements carried out in a homogeneous solution, in micelles, on silica surfaces, inside zeolite pores, or in microcrystalline solids. Tuning of the cage efficiency has also enabled the adaptation of an efficient system for C–H oxygenation to C–H halogenation reactions, as was illustrated by the work of Groves and co-workers on metal oxo porphyrins. Rather than developing a new platform for the generation of a reactive radical from a strong C–H bond, a “simple” reduction in the cage efficiency permits the radical to explore new reaction pathways. In general, cage strengthening is often used to ensure selectivity: selective heterodimerization in N-deletion from secondary amines, selective fragment coupling in total synthesis, protection of radical intermediates from undesired hydrogen-atom abstraction reactions, or retention of stereochemical information encoded in chiral starting materials. Looser cages on the other hand can offer opportunities to transfer a desirable reaction step from one transformation to another or increase reaction efficiency via the possibility of radical chain reactions that proceed via low reaction barriers. For transformations in which radicals are generated via photolysis, the use of shorter wavelength light source can be a facile way of reducing the caging efficiency since the excess energy not required for bond cleavage is given to the radicals in the form of kinetic energy.^156,157^

In many ways, finding ways to exert control over the cage effect could also be likened to the development of the ultimate traceless directing group, because the cage controls the time the molecules it contains spend close to one another. A number of synthetically valuable radical reactions suffer from limitations in their substrate scope because radical addition to carboarenes is more challenging than radical addition to heteroarenes. Similarly, the majority of transition-metal-mediated arene C–H functionalization reactions only proceed in high yields if the arene is either used in solvent quantities or if it carries a directing group. The function of either substrate excess or a metal binding site consists of increasing the time a transition metal catalyst spends in close proximity to the substrate, so that a difficult reaction step has a higher likelihood of taking place. Cage effects have the potential to fulfill a role in transformations proceeding via radical intermediates similar to that played by a directing group in transition metal chemistry: any species sharing a cage with a radical experiences frequent close encounters with the radical, and the likelihood of reaction between the two species is consequently enhanced. The tightness of the cage can be adjusted over a wide range from nonviscous homogeneous solution to porous materials, all the way to transformations taking place in dense solid phases, similar to the ability to tune the concentrating effect of a directing group by varying its affinity for the transition metal catalyst. Since the cage consists of a solvent, a phase boundary, a solid support, or the crystal of the substrate itself, the cage is by its very nature traceless, and advances made in reaction performance based on cage tuning are thus likely to be applicable to a large fraction of potential substrates.

Thus, far, the vast majority of studies on cage effects have focused on the primary cage, in which a pair of radicals is generated. Once a radical escapes from its primary cage, however, its reactions with other closed shell or radical species are once again subject to cage effects as long as the transformation is carried out in a condensed phase. Little is known about these secondary cages, however, and studying them is challenging. The primary solvent cage can be understood through the study of the solvation shell that surrounds a closed shell molecule from which a radical pair is generated. The foundation of the cage effect rests on the idea that little rearrangement of this solvation shell takes place within the first 10^–10^ s after the radicals are formed, so that for the time frame in which it is decided which fraction of radicals undergo either cage escape or recombination, the structure of the solvent cage is known. To better understand the effect of caging on propagation or termination reactions, suitable models need to be devised to permit a systematic study of cages formed when free radicals meet or when radicals and closed shell substrates encounter each other.

## Data Availability

The data underlying this study are available in the published article.
